# Colorful Packages: Encapsulation of Fluorescent Proteins in Complex Coacervate Core Micelles

**DOI:** 10.3390/ijms18071557

**Published:** 2017-07-19

**Authors:** Antsje Nolles, Adrie H. Westphal, J. Mieke Kleijn, Willem J. H. van Berkel, Jan Willem Borst

**Affiliations:** 1 Laboratory of Biochemistry, Wageningen University & Research, Stippeneng 4, 6708 WE Wageningen, The Netherlands; antsje.nolles@wur.nl (A.N.); adrie.westphal@wur.nl (A.H.W.); willem.vanberkel@wur.nl (W.J.H.v.B.); 2 Physical Chemistry and Soft Matter, Wageningen University & Research, Stippeneng 4, 6708 WE Wageningen, The Netherlands; mieke.kleijn@wur.nl; 3 MicroSpectroscopy Centre Wageningen, Wageningen University & Research, Stippeneng 4, 6708 WE Wageningen, The Netherlands

**Keywords:** Anthozoa, chromophore, circular dichroism, diblock copolymer, dynamic light scattering, fluorescence correlation spectroscopy, Hydrozoa, polyelectrolyte, protein structure, steady-state fluorescence

## Abstract

Encapsulation of proteins can be beneficial for food and biomedical applications. To study their biophysical properties in complex coacervate core micelles (C3Ms), we previously encapsulated enhanced green fluorescent protein (EGFP) and its monomeric variant, mEGFP, with the cationic-neutral diblock copolymer poly(2-methyl-vinyl-pyridinium)_n_-*b*-poly(ethylene-oxide)_m_ (P2MVP_n_-*b*-PEO_m_) as enveloping material. C3Ms with high packaging densities of fluorescent proteins (FPs) were obtained, resulting in a restricted orientational freedom of the protein molecules, influencing their structural and spectral properties. To address the generality of this behavior, we encapsulated seven FPs with P2MVP_41_-*b*-PEO_205_ and P2MVP_128_-*b*-PEO_477_. Dynamic light scattering and fluorescence correlation spectroscopy showed lower encapsulation efficiencies for members of the Anthozoa class (*an*FPs) than for Hydrozoa FPs derived from *Aequorea victoria* (*av*FPs). Far-UV CD spectra of the free FPs showed remarkable differences between *av*FPs and *an*FPs, caused by rounder barrel structures for *av*FPs and more elliptic ones for *an*FPs. These structural differences, along with the differences in charge distribution, might explain the variations in encapsulation efficiency between *av*FPs and *an*FPs. Furthermore, the *av*FPs remain monomeric in C3Ms with minor spectral and structural changes. In contrast, the encapsulation of *an*FPs gives rise to decreased quantum yields (monomeric Kusabira Orange 2 (mKO2) and Tag red fluorescent protein (TagRFP)) or to a p*K*_a_ shift of the chromophore (FP variant mCherry).

## 1. Introduction

Fluorescent proteins (FPs) are nowadays indispensable in life sciences [1,2,3,4]. The discovery of FPs started in the early 1960s with studies on the identification of the glow of jellyfish from *Aequorea victoria* by Osamu Shimomura [5]. The protein emitting the green light was called green fluorescent protein (GFP) [6] and its sequence was obtained in 1992 by Prasher [7]. In the following years, a wide variety of GFP variants with different colors and improved brightness and stability were developed. However, there were no GFP variants with emission maxima above 527 nm [4]. This limitation was overcome by cloning of GFP homologs from non-bioluminescent reef corals of the Anthozoa class [8,9,10,11]. From this class, a palette of FPs became available emitting at longer wavelengths. Consequently, the number of applications of FPs has exploded, which is mainly because they can be genetically introduced into cells, tissues or whole organisms. This allows using FPs for multicolor imaging and for studying protein interactions [12,13,14]. Besides using FPs as fusion tags and biosensors, they have also been used as model proteins in encapsulation studies [15,16,17,18].

Encapsulation of proteins is of interest for food and biomedical applications, because it can protect and stabilize the encapsulated protein. Encapsulation of FPs allows the use of fluorescence techniques for the characterization of protein-containing micelles [18].

Previously, we reported on the encapsulation of enhanced green fluorescent protein (EGFP) and its monomeric variant (mEGFP) in complex coacervate core micelles (C3Ms) with the cationic-neutral diblock copolymer poly(2-methyl-vinyl-pyridinium)_n_-*b*-poly(ethylene-oxide)_m_ (P2MVP_n_-*b*-PEO_m_) as enveloping material [18,19]. The two GFP variants showed considerable differences in their spectral and structural properties upon encapsulation. Encapsulation into C3Ms promoted dimerization of EGFP but not of mEGFP, due to the difference in dissociation constant (*K*_D_, 0.11 mM for EGFP and 74.0 mM for mEGFP [20]). Dimerization of EGFP upon encapsulation in C3Ms results in a p*K*_a_ shift of its chromophore, leading to specific changes in the spectral and structural properties of EGFP [19].

**Figure 1:**
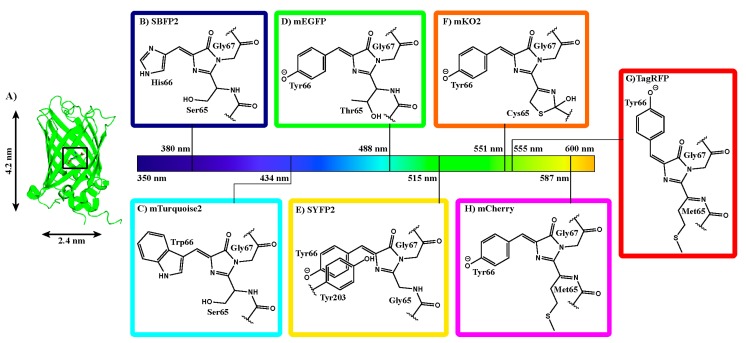
Chromophore properties of fluorescent proteins used in this research (**A**) Ribbon diagram of mEGFP with its chromophore in the center of the barrel (PDB entry 4EUL [29]); (**B**) Chromophore of SBFP2 made from Ser65, His66, and Gly67; (**C**) chromophore of mTurquoise2 made from Ser65, Trp66, and Gly67; (**D**) Chromophore of mEGFP made from Thr65, Tyr66, and Gly67 in the anionic form; (**E**) Chromophore of SYFP2 made from Gly65, Tyr66, and Gly67 in the anionic form with Tyr203 to extend the π-system; (**F**) Chromophore of mKO2 made from Cys65, Tyr66, and Gly67 in the anionic form; (**G**) Chromophore of TagRFP made from Met65, Tyr66, and Gly67 in the anionic form and in the *trans*-conformation and (**H**) Chromophore of mCherry made from Met65, Tyr66 and Gly67 in the anionic form and in the *cis*-conformation. Absorption maxima are indicated on the spectral bar and fluorescence colors are indicated as box colors.

The aim of the present study is to determine whether structural and spectral changes are common upon encapsulation of members of the visible fluorescent protein family. Therefore, we encapsulated a variety of fluorescent proteins covering the whole visible spectrum (Figure 1). We investigated seven differently colored FPs: four FPs derived from *Aequorea victoria* GFP (*av*FPs: strongly enhanced blue fluorescent protein 2 (SBFP2), mTurquoise2, mEGFP, and strongly enhanced yellow fluorescent protein 2 (SYFP2), from class Hydrozoa) and three FPs from class Anthozoa (*an*FPs: monomeric Kusabira Orange 2 (mKO2), Tag red fluorescent protein (TagRFP), and mCherry). The seven FPs originate from four different ancestors: one from the Hydrozoa class and three from the Anthozoa class. Even though these proteins have a comparable fold and function, the amino acid sequences vary between the different species (see Figure S1 and Table S1). The sequence identities among the *av*FPs are about 97%, whereas the *an*FPs show about 25–30% similarity to the *av*FPs. Amongst the *an*FPs, TagRFP and mCherry show a higher sequence identity (57%) than mKO2 does with these two proteins (~48%). The seven FPs contain about 10% of strictly conserved residues (Figure S1). These residues are mainly located at the end of beta-strands and in loops, except for the strictly conserved Gly67, Asp95, Arg96, and Glu222 amino acid residues, which are involved in the formation of the chromophore [21].

Some general properties of FPs are critical for understanding their structural and spectral properties. All FPs have an approximate molar mass of 27 kDa, constituting a single polypeptide chain of about 230 amino acids. They all share a fold consisting of an 11-stranded β-barrel with a length of 4.2 nm and a diameter of 2.4 nm (Figure 1A). The barrel is wrapped around a single distorted helix, which contains three amino acid residues that create the fluorophore. The fluorophore is formed via cyclization, dehydration, and oxidation of the amino acid residues located at positions 65–67 (mEGFP numbering, Figure 1B–H). Depending on the pH of the solution, the chromophore can exist in differing protonation states, which influences the spectral properties of the FP. Furthermore, the fluorophore is comprised of a highly conjugated π-electron resonance system that together with its environment accounts for the spectroscopic and photophysical properties of an FP.

The spectroscopic features of the seven FPs will be presented according to their absorption maxima. We start with strongly enhanced blue fluorescent protein (SBFP2), which was obtained by the Y66H substitution in GFP (Figure 1B) [22]. Turquoise FPs are obtained by a Y66W substitution in GFP (Figure 1C), which yields amongst others mTurquoise2 [23]. mTurquoise2 is especially characterized by its long mono-exponential fluorescence lifetime (τ = 3.8 ns), which makes it very suitable as a Förster resonance energy transfer (FRET) donor in conjunction with a yellow fluorescent protein as the acceptor. Originally, FPs are not monomeric, but this can be achieved with the A206K substitution, which is used in mEGFP [20]. The GFP variants with emission maxima at the longest wavelengths are yellow FPs, obtained by a T203Y substitution in GFP, and in this research we used SYFP2 (Figure 1E) [24]. This FP has a high extinction coefficient compared to other FPs (ε_SYFP2_ = 101 000 M^−1^·cm^−1^, see Table S2) making it a very suitable acceptor in FRET-pairs [24].

The chromophore structures of FPs from Anthozoa species generally have more extended π-systems, enabling higher excitation and emission wavelengths. Such a type of fluorophore is found in mKO2, which evolved from a fluorescent protein of the mushroom coral *Fungia concinna*, with a cysteine located at position 65 (mEGFP numbering, Figure 1F) [9,25]. mKO2 is very useful in multicolor imaging applications as it can be combined with cyan, green, yellow, and red FPs. A protein with an almost similar excitation maximum as mKO2 is TagRFP (Figure 1G), but this protein has been derived from the sea anemone *Entacmaea quadricolor* [10]. TagRFP has an even more extended π-system than mKO2, because it has a methionine located at position 65 (mEGFP numbering, Figure 1G). Next to that, TagRFP is one of the few FPs bearing a *trans*-isomerized chromophore. A protein that also contains a methionine at position 65 (mEGFP numbering) is mCherry, one of the “mFruit” FPs derived from *Discosoma species* [26,27]. mCherry shows a high photostability and its chromophore is rapidly formed (Figure 1H), which makes it very suitable as a FRET acceptor in combination with EGFP in fluorescence-lifetime imaging microscopy (FLIM) studies [28].

In this research, we used the diblock copolymers P2MVP_41_-*b*-PEO_205_ and P2MVP_128_-*b*-PEO_477_ to form C3Ms in combination with the above-mentioned FPs. We characterized the C3Ms with dynamic light scattering (DLS) and fluorescence correlation spectroscopy (FCS), and explored the effects of packing on the FPs with circular dichroism (CD) and fluorescence spectral analysis. The experimental data, and in particular the observed encapsulation efficiencies, are discussed in relation to what is known about the structural features of the FPs.

## 2. Results

**Figure 2:**
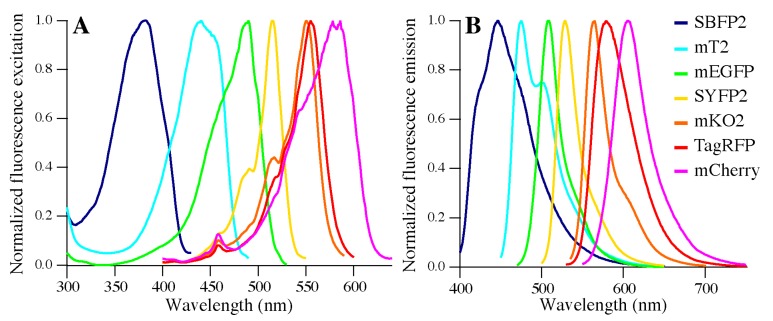
Normalized fluorescence (**A**) Excitation spectra and (**B**) Emission spectra of SBFP2, mTurquoise2 (mT2), mEGFP, SYFP2, mKO2, TagRFP and mCherry.

In this work, we purified seven FPs using either the intein/chitin-binding-domain system (for mTurquoise2, SYFP2-His, and mCherry) or metal affinity chromatography (for SBFP2, mEGFP, mKO2, and TagRFP, see Section 4.2). The influence of the His-tag was tested by encapsulation of mTurquoise2 and of mTurquoise2-His [30]. We did not observe any differences in encapsulation properties between the two proteins; therefore, it is presumed that His-tags have no effects on our experiments using other FPs. The purified FPs have distinctive spectral properties. Figure 2 shows our recorded normalized fluorescence excitation and emission spectra of the FPs, which display maxima in agreement with those listed in literature (Table S2).

### 2.1. Fluorescent Protein Charge Determination

Measurements on encapsulated FPs in C3Ms are commonly performed at the preferred micellar composition (PMC), which is the ratio between protein and polymer at which the highest concentration of micelles is obtained [18,19,31]. The PMC is defined in terms of the total concentration of positively charged groups on the polymers and the net concentration of negative charges on the protein (see Equation (1), Section 4.5). The positive charge on the polymers is fixed due to quaternization, but the charge of the proteins varies with pH. The amino acid residues on the protein surface determine to a great extent the net charge of the protein, which can be deduced from the protein’s three-dimensional structure using the PROPKA software package [32,33]. For four of the studied FPs (mTurquoise2, mEGFP, TagRFP, and mCherry), a crystal structure is available in the RCSB Protein Data Bank [34] (Table 3). For the three other FPs (SBFP2, SYFP2, and mKO2), a Blast search was performed to obtain the most suitable template, which was then used for building homology models (Table 4).

The p*I* value for the four *av*FPs and for two *an*FPs (mKO2 and mCherry) is about 5.5, while TagRFP has a significantly higher p*I* value, ~7.6 (Table S2). To achieve similar electrostatic interactions between the polymers and the different FPs, we encapsulated all FPs at the pH at which they have a net negative charge of about 10 unit charges. Thus, TagRFP was encapsulated at pH 10 and the other FPs at pH 9 (Table 1). At these conditions, all FPs are stable [35].

### 2.2. Preferred Micellar Composition (PMC)

**Figure 3:**
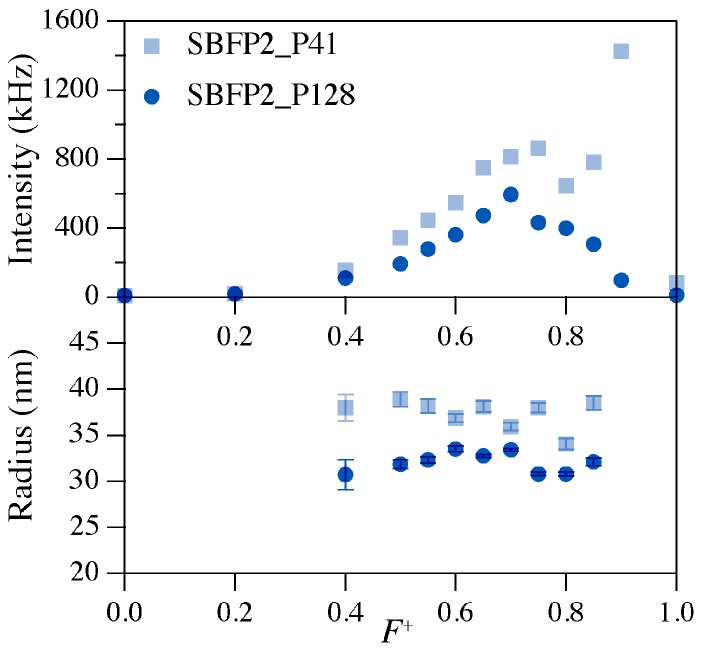
DLS results of micellar compositions of SBFP2 mixed with P2MVP_41_-*b*-PEO_205_ (P41, light blue colored blocks) or P2MVP_128_-*b*-PEO_477_ (P128, dark blue colored circles). The protein concentration was kept constant at 1.0 μM. Top graph shows scattered intensity as a function of the *F*^+^ composition, and bottom graph shows the hydrodynamic radius as a function of the *F*^+^ composition. Error bars reflect the distribution of radii in one experiment. DLS composition results of the six other fluorescent proteins are given in Figure S2.

The seven FPs were encapsulated using two diblock copolymers with different lengths (P2MVP_41_-*b*-PEO_205_ or P2MVP_128_-*b*-PEO_477_). As a start, dynamic light scattering (DLS) experiments were performed to determine the PMCs. The results of SBFP2 with P2MVP_41_-*b*-PEO_205_ and P2MVP_128_-*b*-PEO_477_ are shown in Figure 3. The highest concentration of micelles is found at the maximum of the scattered light intensity. For SBFP2, PMCs are found at *F*^+^ values of 0.75 and 0.70 for P2MVP_41_-*b*-PEO_205_ and P2MVP_128_-*b*-PEO_477_, respectively (Figure 3 and Table 1). Similar DLS experiments were performed on the other six FPs with both diblock copolymers (Figure S2) and their respective PMCs are listed in Table 1. For all FPs and with both diblock copolymers, optimal *F*^+^ values ranging between 0.60 and 0.75 were found. Samples with this optimal composition were used in all other spectroscopic analyses: fluorescence correlation spectroscopy (FCS), circular dichroism (CD), and steady-state fluorescence spectroscopy.

The fluctuations of the scattered light intensities were used to calculate the hydrodynamic radii of the C3Ms. For all seven FPs, the hydrodynamic radii of the C3Ms are quite constant over a relatively wide range of *F*^+^ compositions (0.40 < *F*^+^ < 0.80, Figure 3 and Figure S2). In general, radii of the formed C3Ms vary between 30 and 38 nm, except for C3Ms formed with mKO2, which are somewhat smaller with radii of about 27 nm (Table 1).

### 2.3. Encapsulation Efficiency

**Figure 4:**
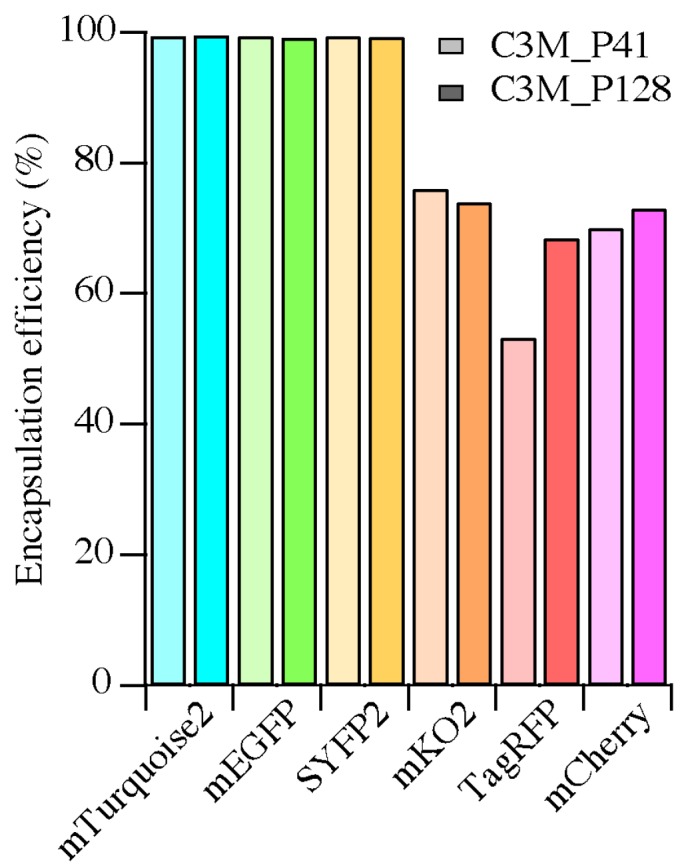
Encapsulation efficiencies of the different fluorescent proteins studied (except SBFP2) at their respective PMCs with P2MVP_41_-*b*-PEO_205_ (C3M_P41, light colored bars) and P2MVP_128_-*b*-PEO_477_ (C3M_P128, dark colored bars) determined using FCS. Efficiencies are calculated from the average number of fluorescent particles observed in the confocal volume (*N* in Equation (2) and Figure S3).

Next to DLS, FCS can be used for the determination of PMC values [18]. An advantage of FCS is that it gives, amongst other parameters, the average number of fluorescent particles in the confocal volume (*N*, Equation (2)). In this study, the fluorescent particles observed are free FPs and C3Ms with multiple FPs encapsulated. We quantified the number of free FPs before addition of polymers (*N*_before_) and of fluorescent particles after addition of polymers (*N*_after_), and expressed the encapsulation efficiency per FP according to the following relation: *E*_encap_ = 1 − (*N*_after_/*N*_before_). The encapsulation efficiencies per FP are shown in Figure 4 and the corresponding graph with the number of fluorescent particles is shown in Figure S3. FCS was not performed on samples containing SBFP2 because no suitable excitation source for this FP was available on the used confocal microscope.

For all *av*FPs, the encapsulation efficiencies are almost 100% with both diblock copolymers, meaning that virtually all protein molecules are packed in C3Ms. However, we observed lower encapsulation efficiencies for *an*FPs (50% to 75%, see Figure 4), which implicates that, for these FPs, more protein molecules remain free in solution (Figure S3).

### 2.4. Fluorescence Properties

**Figure 5:**
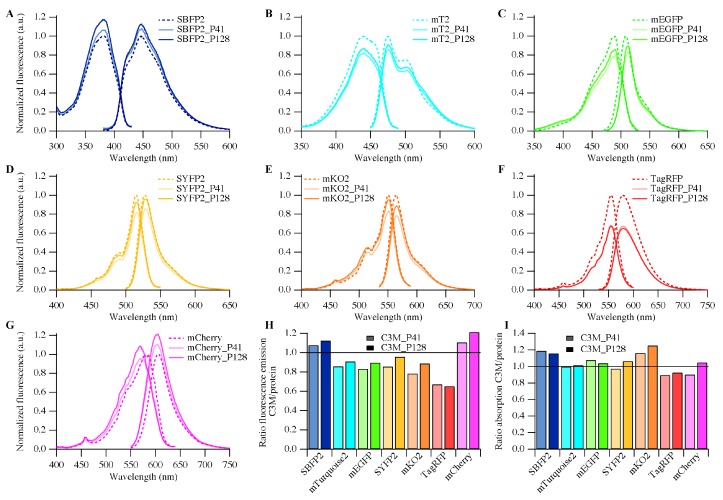
Fluorescence excitation and emission spectra of (**A**) SBFP2; (**B**) mTurqouise2; (**C**) mEGFP; (**D**) SYFP2; (**E**) mKO2; (**F**) TagRFP and (**G**) mCherry for protein free in solution (dashed lines) and encapsulated proteins in C3Ms at their respective PMCs with P2MVP_41_-*b*-PEO_205_ (P41, solid light colored lines) and P2MVP_128_-*b*-PEO_477_ (P128, solid dark colored lines). The spectra are normalized to those of the free proteins measured at identical conditions. (**H**) Ratio of fluorescence emission; and (**I**) ratio of absorption of encapsulated protein (C3M) to that of protein free in solution (values for mCherry are taken from the maxima). Ratios for all proteins were measured at the PMC with P2MVP_41_-*b*-PEO_205_ (C3M_P41, light colored bars) and P2MVP_128_-*b*-PEO_477_ (C3M_P128, dark colored bars). The corresponding absorption spectra are displayed in Figure S4.

Previously, we have shown that encapsulation of EGFP and mEGFP resulted in different spectral properties compared to that of the proteins free in solution [19]. The spectral properties of EGFP upon encapsulation do not changes more than that of mEGFP, which is due to the p*K*_a_ shift of the chromophore of EGFP. To investigate if encapsulation changes the spectral properties of the FPs, absorption and fluorescence excitation and emission spectra for all FPs free in solution, as well as encapsulated in C3Ms were recorded (Figure 5 and Figure S4).

We observed that encapsulation of the FPs leads to minor differences in their absorption and fluorescence properties and these are dependent on the kind of FP and the type of polymer used. Figure 5H,I shows that, for SBFP2, both the absorption and the fluorescence intensity increases upon encapsulation. Encapsulation of mTurquoise2, mEGFP, and SYFP2 resulted in a decrease of the fluorescence intensity, whereas the absorption remained the same. Both the absorption and fluorescence intensity decreases upon encapsulation of TagRFP. For mCherry, the fluorescence intensity increases and the absorption and excitation maxima become blue-shifted upon encapsulation (for absorption spectra see Figure S4). The absorption and fluorescence results were combined in the determination of relative quantum yields of FPs encapsulated in C3Ms (Equation (4) and Table 2). Table 2 shows that the quantum yield of SBFP2 does not change; that of mCherry increases; and that of the other FPs decreases upon encapsulation.

To address if the observed spectral changes are due to a pH-related phenomenon, fluorescence excitation and emission spectra at different pH values were acquired of all FPs free in solution (see Figure S5). SBFP2, mEGFP, SYFP2, and mKO2 have a p*K*_a_ of 5.5–6.0 and show a large decrease in their fluorescence intensity at pH 5. For the latter three proteins, this effect is caused by protonation of the phenolic oxygen of the chromophore (Figure 1D–F and Figure S5C–E). mCherry shows a stronger susceptibility to changes in pH (Figure S5G): at increasing pH values (from pH 5 to 10), the spectra are blue-shifted and the fluorescence intensity increases. These changes resemble the changes observed upon encapsulation of mCherry.

The only two FPs showing no significant effect upon changes of pH are mTurquoise2 and TagRFP, which can be explained by their rather low p*K*_a_ values (p*K*_a_ ~3.5, see Figure S5F). It is therefore remarkable that the fluorescence intensity of TagRFP decreases about 40% upon encapsulation compared to the free protein (Figure 5H), even though the encapsulation efficiency is about 60% (Figure 4). This suggests that the fluorescence of TagRFP is highly affected upon encapsulation. In solution, TagRFP tends to dimerize with a *K*_D_ of 38.4 μM [36]. Assuming a protein concentration of about 10 mM in the C3Ms, this implies that TagRFP associates into dimers or tetramers inside C3Ms, which might cause the drastic decrease of quantum yield of the chromophore upon encapsulation.

Next to these differences between the FPs, we also observed an effect depending on the length of diblock copolymer used: if the fluorescence increases upon encapsulation, the increase is larger with the longer polymer (P2MVP_128_-*b*-PEO_477_) than with the shorter one (P2MVP_41_-*b*-PEO_205_). Conversely, if the fluorescence decreases upon encapsulation, the decrease is larger with the shorter polymer than with the longer one, except for TagRFP (Figure 5H). This dependency, however, is not observed in the absorption spectra (Figure 5I).

### 2.5. Secondary Structure

**Figure 6:**
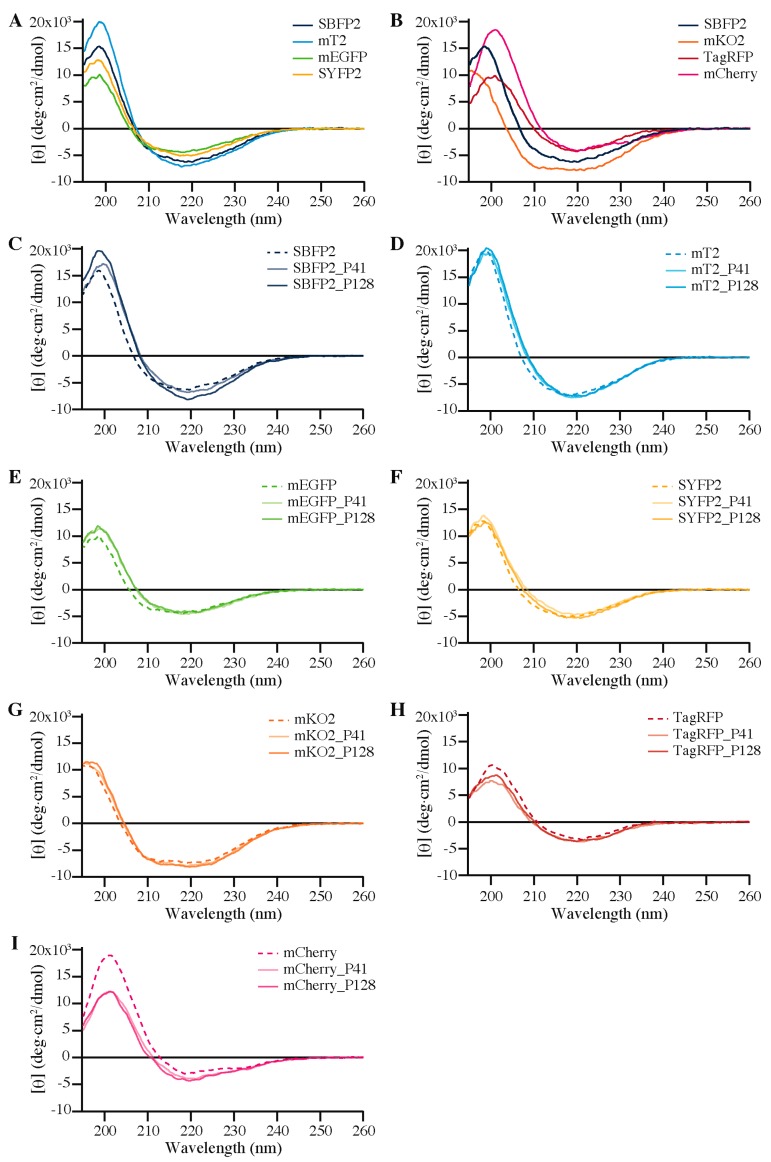
Comparison of the far-UV CD spectra of the free proteins (**A**) *av*FPs; (**B**) *an*FPs with SBFP2 as a reference representing the *av*FPs. Far-UV CD spectra of (**C**) SBFP2; (**D**) mTurquoise2 (mT2); (**E**) mEGFP; (**F**) SYFP2; (**G**) mKO2; (**H**) TagRFP and (**I**) mCherry free in solution (dashed line) and encapsulated with P2MVP_41_-*b*-PEO_205_ (P41, solid light colored line) and P2MVP_128_-*b*-PEO_477_ (P128, solid dark colored line). The corresponding high tension signals are shown in Figure S6.

To investigate whether the differences in encapsulation efficiencies are due to structural perturbations of the FPs, far-UV circular dichroism (CD) experiments were performed. Figure 6 shows CD spectra of all seven FPs free in solution and encapsulated with P2MVP_41_-*b*-PEO_205_ or with P2MVP_128_-*b*-PEO_477_. The CD spectra of the FPs are not affected by the increase in pH, as the CD spectra at pH 9.0 or 10.0 do not show any differences compared to those at pH 7.0 [37].

For all FPs, a negative mean residue ellipticity near 220 nm was observed, which is in good agreement with the prominent β-barrel architecture of these proteins (Figure 1A) and in line with previous observations [19,38]. The spectrum of mKO2, however, resembles more a α-helical architecture with two negative peaks near 210 and 220 nm [39,40].

The CD spectra of the four *av*FPs free in solution are quite similar in shape (Figure 6A). The CD spectra of the *an*FPs are remarkably different compared to those of the *av*FPs (SBFP2 was taken as a representative reference, see Figure 6B). To our knowledge, these differences have not been reported before, and are further addressed in Section 3.2.

Upon encapsulation of the FPs, the CD spectra alter to a greater or lesser extent compared to that of the proteins free in solution, especially in the range where the spectra switch ellipticity (“zero crossing”, between 205 and 215 nm). For the encapsulated *av*FPs and for encapsulated mKO2, the zero crossing shifts to higher wavelength compared to that of the respective free FPs (Figure 6C–G). On the other hand, the zero crossings of encapsulated TagRFP and mCherry change to lower wavelengths compared to that of the free proteins (Figure 6H,I). In general, the zero crossings of all encapsulated FPs shift to ±210 nm. Apart from the zero crossings, the changes upon encapsulation of mTurquoise2, SYFP2, and mKO2 are moderate. More pronounced deviations in CD spectra after encapsulation are observed for SBFP2, mEGFP, and TagRFP. The largest change, however, can be observed for mCherry, with a significant positive decrease and a negative increase in ellipticity around 200 and 220 nm, respectively (Figure 6I).

## 3. Discussion

Previously, we found that the encapsulation of EGFP in C3Ms stimulates protein dimerization and changes the spectral properties of the EGFP chromophore [19]. Because mEGFP mainly remains monomeric in the densely packed C3Ms, encapsulation of this protein hardly affects its spectral properties. In this work, we studied the encapsulation of four *av*FPs and three *an*FPs in C3Ms. All investigated FPs were successfully encapsulated using two diblock copolymers (P2MVP_41_-*b*-PEO_205_ and P2MVP_128_-*b*-PEO_477_) with *F*^+^ values ranging between 0.60 and 0.80. For strong polyelectrolytes, stoichiometric C3M systems are formed at a *F*^+^ value of 0.50 [41]. Proteins, however, are weak polyelectrolytes and therefore their charge may change upon interaction with the diblock copolymer. Moreover, coacervation between polymer and protein does not necessarily arise from the overall charge of the protein, but rather from specific charge patches on the protein surface [42]. Both effects can even lead to coacervation between similarly charged proteins and polyelectrolytes [43,44,45,46].

### 3.1. Encapsulation Efficiency

**Figure 7:**
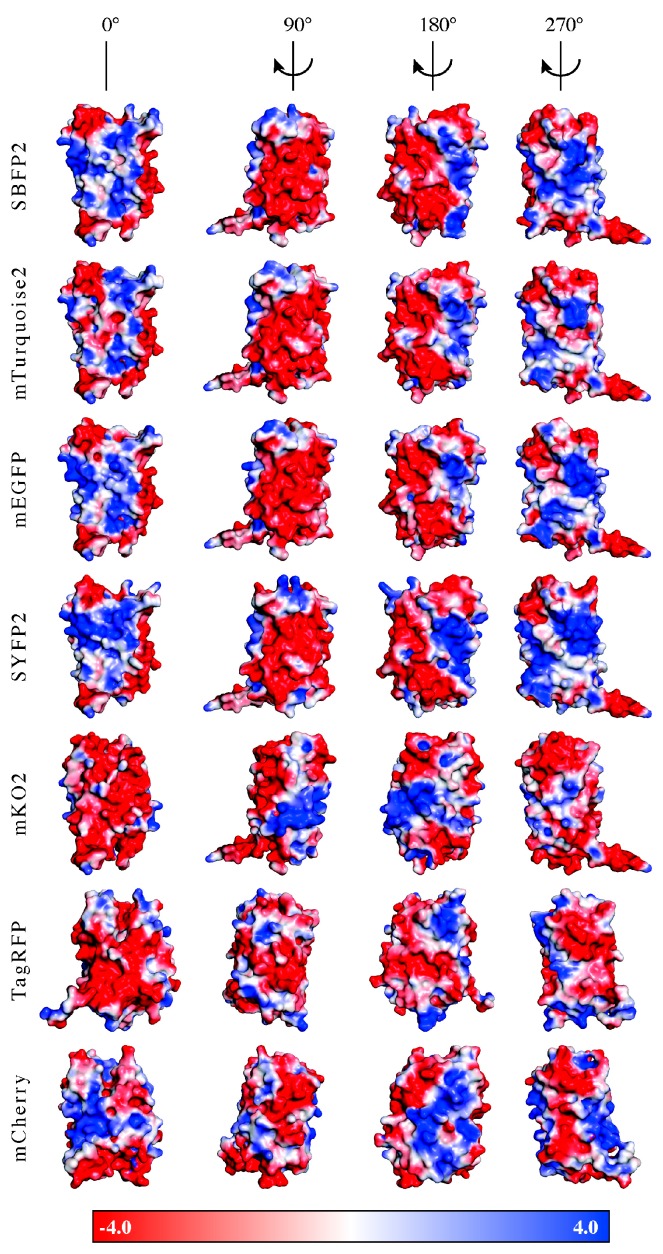
Comparison of the seven investigated proteins’ electrostatic potentials (in *k_B_T*/*e_c_*) at the molecular surface in four different orientations: SBFP2, mTurquoise2, mEGFP, SYFP2, mKO2, TagRFP, and mCherry. Color surface overlay denotes electrostatic potential according to the scale shown: Red, negative potential; white, neutral; and blue, positive potential. Figure created by solution of the Poisson–Boltzmann equation using the default parameters of the PyMOL APBS (Adaptive Poisson-Boltzmann Solver) Tools plugin [49,50] in MacPyMOL 1.4 [51].

The encapsulation efficiencies of *av*FPs (mEGFP, SBFP2, SYFP2 and mTurquoise2) were almost 100%, whereas those of *an*FPs (mKO2, TagRFP and mCherry) varied between 50% and 75%. This implicates that the interactions between the *an*FPs and the diblock copolymers to form C3Ms are less favorable. The formation of C3Ms requires an interaction between the FPs and the polymers, which can be dependent on the surface charge distribution and/or the shape of the protein. For the investigation of the presence of specific charge patches on the protein surface, we determined the surface potential distribution of the FPs on the acquired protein structures. For this, homology modeling was used to obtain the protein structures of SBFP2, SYFP2, and mKO2, next to the crystal structures of mTurquoise2, mEGFP, TagRFP, and mCherry. In Figure 7, the surface potentials of the FPs are visualized at the pH value at which they were encapsulated. All *av*FPs share a negative surface patch, as displayed on the side view at 90°, with an expansion to half of the molecule displayed in the side view at 180°. The amino acid residues with negative charge belonging to this patch are located on β-strands 1 and 2. The three *an*FPs do not contain a similar negative patch displayed on the side view at 90°, as observed for *av*FPs. Negative patches for mKO2 and TagRFP are mainly present in the side view at 0°. For TagRFP, the amino acid residues with negative charge are more distributed over the entire protein surface than for the other proteins. For mCherry, there is not a side entirely filled with negatively charged amino acid residues. It is key for the positively charged polyelectrolyte to bind to a local negative charge patch on the protein while minimizing the repulsive effect arising from the positively charged amino acid residues. Therefore, the interactions between the diblock copolymers and mKO2, TagRFP, and mCherry might not be optimal, thus affecting their encapsulation efficiencies.

### 3.2. Elliptical Symmetry of FP Barrels

**Figure 8:**
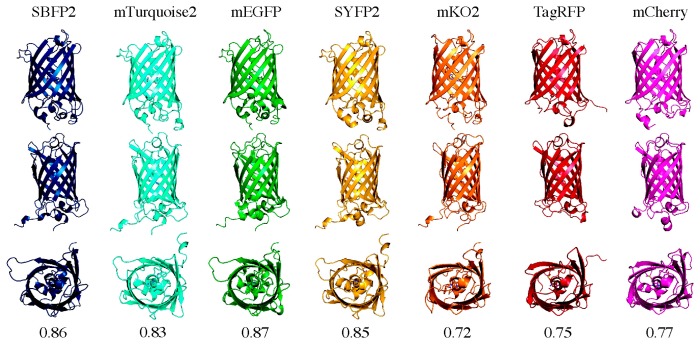
Ribbon structures of the seven FPs in three different orientations. At the top, the broad side; in the middle, the narrow side; and at the bottom, the “top” (the base containing both termini) of the proteins is shown. Numbers indicate the aspect ratio between the narrow and the broad side of the proteins.

During this study, we uncovered clear differences in the far-UV CD spectra between *av*FPs and *an*FPs free in solution (Figure 6B). It is well known that all FPs share a similar 11-stranded β-barrel fold (Figure 1A). However, it is hardly reported in the literature that the elliptical symmetry between *av*FPs and *an*FPs is diverse [47]. Figure 8 shows the ribbon structures of the studied FPs in three different orientations: the broad side, the narrow side, and the top. From the top views, it is clear that the barrels of the FPs are not completely round, but form elliptic cylinders. The *av*FPs are rounder than the *an*FPs, which is depicted by the difference in aspect ratio: ~0.85 for the *av*FPs and ~0.74 for the *an*FPs. Especially mKO2 is the most “squeezed” of the *an*FPs. We hypothesize that these differences are the cause for the observed differences in the far-UV CD spectra. Micsonai et al. reported that CD spectra are influenced, among others, by degree of twist and distortion of the β-sheets [48]. The variance in the elliptical symmetry is another apparent difference between *av*FPs and *an*FPs, and could also be influencing their encapsulation efficiencies.

### 3.3. Biophysical Properties of Encapsulated Proteins

Encapsulation of the *av*FPs hardly influenced their secondary structural properties and only minor changes in absorption and emission characteristics were observed. All *av*FPs bear the A206K mutation, which favors their monomeric state. This supports that the minor spectral changes observed are caused by the electrostatic interactions between the polymers and the protein surfaces of these FPs.

All *an*FPs are found as tetramers in their hosts [9,10,26]. The *an*FPs used here are all modified to enhance their tendency to remain monomeric. In literature, this tendency is expressed in terms of dissociation constants and “monomeric quality” (see Table S2). Previously, we calculated the number of EGFP molecules present in a C3M to be around 400, yielding a local protein concentration of about 10 mM [18,19]. Since the FPs used in this study form C3Ms with PMCs (~0.65) and radii (~34 nm) similar to EGFP-C3Ms, it is a reasonable assumption that the protein concentration in the various FP-C3Ms is about the same. Hence, we expect that mCherry with a monomeric quality of 95% remains mostly monomeric upon encapsulation. However, mKO2 and TagRFP with monomeric qualities of 68% and 58%, respectively, and a dissociation constant of 0.038 mM for TagRFP, will likely form oligomers in the C3Ms (Table S2). This oligomerization causes the large decrease in quantum yield of the encapsulated forms of mKO2 and TagRFP (Table 2).

For encapsulated mCherry, the absorption spectrum changes according to a p*K*_a_ shift of its chromophore (Figure S4). For EGFP it was proposed that the p*K*_a_ shift of its chromophore is caused by a reorientation of Glu222 due to the dimerization of EGPF in C3Ms [19]. For free mCherry, the equivalent Glu215 is also linked to the pH-dependent spectral shifts (Figure S5G) [27]. If mCherry, however, remains monomeric in the C3Ms, the reorientation of Glu215 can only occur due to the interaction between protein and polymer.

### 3.4. Future Research

We show that encapsulation of structurally similar FPs in C3Ms is dependent on the origin of the FPs and can give rise to different encapsulation efficiencies. Moreover, the spectral and structural perturbations observed are dependent on the kind of FP and the type of polymer used. In future research, we plan to investigate the stability and dynamics of encapsulated FPs. This can be accomplished by mixing two appropriate FPs using FRET as a readout. Some requirements should be considered choosing an optimal FP FRET-pair: First, use fluorescent proteins with similar encapsulation efficiencies. Second, use FPs that show minor changes in their absorption and fluorescence properties upon encapsulation into the C3Ms. Third, use the diblock copolymer which has the least effect on the fluorescence properties of the FPs. According to the present results, the ideal partners of an FRET-pair in C3Ms would be mTurquoise2 and SYFP2 in combination with P2MVP_128_-*b*-PEO_477_.

## 4. Materials and Methods

### 4.1. Materials

Poly(2-vinyl-pyridinium)_n_-*b*-poly(ethylene-oxide)_m_ (P2VP_n_-*b*-PEO_m_) with different chain lengths was quaternized: P2VP_41_-*b*-PEO_205_ (Polymer Source *Inc.*, Dorval, Quebec, Canada, *M_w_*/*M_n_* = 1.05, *M_n_* = 13.3 kg/mol) and P2VP_128_-*b*-PEO_477_ (Polymer Source *Inc.*, Canada, *M_w_*/*M_n_* = 1.10, *M_n_* = 34.5 kg/mol), following a procedure described elsewhere [18]. For P2MVP_41_-*b*-PEO_205_ (*M_n_* = 18.6 kg/mol) and for P2MVP_128_-*b*-PEO_477_ (*M_n_* = 50.8 kg/mol) a final degree of quaternization of approximately 80% and 87% was obtained, respectively. Stock solutions of P2MVP_41_-*b*-PEO_205_ (51 μM) and P2MVP_128_-*b*-PEO_477_ (50 μM) were prepared by dissolving the polymers in 10 mM borate buffer (pH 9.0) and stored at −20 °C. All solutions were filtered through 0.20 μm polyethersulfone membrane syringe filters (Advanced Microdevices Pvt. Ltd., Ambala Cantt, India). All other chemicals were from commercial sources and of the purest grade available.

### 4.2. Protein Production

The cDNA’s coding for mTurquoise2, SYFP2-His, and mCherry were cloned into the bacterial expression vector pTYB12 (New England Biolabs, Ipswich, MA, USA) to generate FP fusions with a chitin-binding domain and an intein [52,53,54]. The cDNA’s of SBFP2, mEGFP, mKO2, and TagRFP in pRSETb vectors were kindly provided by Dr. J. Goedhart, University of Amsterdam [22,23,24]. For protein production, *E. coli* BL21 cells were used. Details on protein production and purification are described elsewhere [19]. FPs without the chitin-binding domain were acquired after on-column cleavage of mTurquoise2, SYFP2-His, and mCherry. The other FPs, i.e., SBFP2, mEGFP, mKO2, and TagRFP still contained the His-tag after purification. Purified protein samples were stored in 10 mM borate buffer (pH 9.0) at −20 °C.

Protein concentrations were determined with a Pierce BCA protein assay (Pierce Biotechnology, Rockford, IL, USA), using a bovine serum albumin standard as a reference. The purity of the FPs was checked by SDS-PAGE.

### 4.3. Modeling

Homology models were built from existing crystal structures using SWISS-MODEL [55,56,57,58]. Table 3 shows the proteins used in this paper and their corresponding PDBs. Table 4 shows the proteins used in this paper and their respective templates used for the homology modeling. The chromophores were placed in the model structure at the same position and orientation as the chromophore in the template structure. Pairwise sequence alignments of the FPs are listed in Figures S7–S13. Because some N- and C-termini were missing in the created homology models (for SBFP2, mEGFP, and SYFP2), these termini were modeled manually using the PDB entry 3ZTF as a template. The A206K mutants were created by mutagenesis of Ala206 into Lys206 in PDB entries 4EUL and 3ZTF to construct mEGFP and mTurquoise2, respectively.

**Table 3:** PDB structures used for the proteins studied in this research listed with their corresponding figures, sequence identities (% ID) and references.

Protein	PDB Entry	Figure	% ID	Reference
mTurquoise2	3ZTF	S8	99.57	Goedhart, et al. [23]
mEGFP	4EUL	S9	99.56	Arpino, et al. [29]
TagRFP	3M22	S12	100.00	Subach, et al. [59]
mCherry	2H5Q	S13	100.00	Shu, et al. [27]

**Table 4:** Homology models built from PDB entry templates for the proteins studied in this research listed with their corresponding figures, sequence identities (% ID) and references.

Protein	PDB Entry	Figure	% ID	Reference
SBFP2	1BFP	S7	96.44	Wachter, et al. [60]
SYFP2	1MYW	S10	99.12	Rekas, et al. [61]
mKO2	2ZMU	S11	95.31	Kikuchi, et al. [62]

### 4.4. C3M Preparation

Encapsulation of FPs with polymers was achieved by first diluting the FP stock solution in 10 mM borate buffer at pH 9.0 for SBFP2, mTurquoise2, mEGFP, SYFP2, mKO2, and mCherry; and at pH 10.0 for TagRFP to the desired concentration, followed by addition of the polymer. After mixing, samples were stored at room temperature for 24 h before measuring. All experiments were performed in 10 mM borate buffer at the encapsulation pH.

### 4.5. Dynamic Light Scattering (DLS)

DLS measurements were performed on an ALV instrument equipped with a 300 mW Cobolt Samba-300 DPSS laser operating at 660 nm and 100 mW, and static and dynamic enhancer fiber optics for an ALV/High QE APD (high quantum efficiency avalanche photo diode) single photon detector connected to an ALV5000/60X0 External Correlator (ALV-Laser Vertriebsgesellschaft m-b.H., Langen, Germany). The detection angle *θ* was set at 90° and all measurement were performed at room temperature.

DLS measures fluctuations in scattered light intensities caused by the diffusion of particles. The diffusion time of particles is dependent on their size: proteins diffuse faster than the encapsulated proteins in C3Ms. Furthermore, larger particles scatter more light, because the scattered light intensity is proportional to *R*^6^, where *R* is the particle radius. The formation of more C3Ms leads to higher light intensities, which results in a maximum in the scattered light intensity versus composition plot (*I* vs. *F*^+^). The composition at the maximum in scattered light intensity is denoted as the preferred micellar composition (PMC). For determination of the PMC, 500 μL solutions with different polymer/protein compositions were prepared. The protein concentration was kept constant at 1 μM for each composition. The amount of P2MVP_41_-*b*-PEO_205_ or P2MVP_128_-*b*-PEO_477_ was varied to obtain the desired values of *F*^+^:

(1) $$F^{+}=\frac{\left[n_{+}\right]}{\left[n_{+}\right]+\left[n_{-}\right]}$$

where [*n*_+_] = *c_+_N_+_* refers to the total concentration of positively charged groups on the polymer and [*n*_−_] = *c_−_N_−_* is the total concentration of negatively charged groups on the protein molecules. The number of charged groups on the diblock copolymer (*N*_+_) taking the degree of quaternization into account, is +33.1 for P2MVP_41_-*b*-PEO_205_ and +112.0 for P2MVP_128_-*b*-PEO_477_, which is used to calculate [*n*_+_]. The net charge of the proteins as a function of pH was calculated using the software package PROPKA 3.1 [32,33]. The charges of the native proteins at pH 9 or 10 (*N*_−_) are listed in Table 1, which are used to calculate [*n*_−_].

### 4.6. DLS Data Analysis

DLS autocorrelation curves were generated from 10 intensity traces and averaged. The CUMULANT method [63,64] was used to analyze the mean apparent hydrodynamic radius (*R*_h_) as:

(2) Rh=kT q26πηΓ

where *q* is the scattering vector, *k* is the Boltzmann constant, *T* is the absolute temperature, *n* is the viscosity of the solvent, and *Γ* is the measured average decay rate of the correlation function. The CONTIN method [65,66] is used to analyze the distribution of the radii of the C3Ms. The data were analyzed with the AfterALV program (AfterALV 1.0d, Dullware, The Netherlands).

### 4.7. Fluorescence Correlation Spectroscopy (FCS)

FCS was performed on a Leica TCS SP8 X SMD system equipped with a 63× 1.20 NA (numeric aperture) water immersion objective with coverslip thickness correction collar. Samples with FPs were excited using a diode laser (emits at 440 nm) or a super continuum laser (emits a continuous spectrum from 470 to 670 nm). The lasers were set at a pulsed frequency of 40 MHz. The size-adjustable pinhole was set at 70 μm for all measurements. Fluorescence emission was detected using bandpass-adjustable spectral filters. In Table 5 the used laser lines and range of the spectral filters are given per fluorescent protein. Fluorescence was recorded via the internal hybrid detector, which was coupled to a PicoHarp 300 TCSPC module (PicoQuant, Berlin, Germany). With this system, it was not possible to measure SBFP2, because its excitation maximum is below 440 nm.

Rhodamine 110 (*D* = 4.3 × 10^−10^ m^2^ s^−1^) was used to calibrate the confocal volume of the setup. A diffusion time of 18 μs and a structural parameter (*a*, expressed as (ω_xy_/ω_z_)) between 5 and 10 were obtained, resulting in a confocal volume of approximately 0.2 fL. Measurements were performed in a μ-Slide 8-wells chambered coverslip (Ibidi^®^).

Samples with concentrations of 1 μM FP were measured free in buffered solution as well as encapsulated with P2MVP_41_-*b*-PEO_205_ or P2MVP_128_-*b*-PEO_477_ at their respective PMCs. For each sample, 5 fluorescence intensity fluctuation traces of 30 s each were collected. All measurements were performed at room temperature.

### 4.8. FCS Data Analysis

For the FCS data analysis, the FFS-data processor version 2.3 (Scientific Software Technologies Software Centre, Minsk, Belarus) was used [67]. The equation used to fit translational data, which includes triplet state, is as follows [68]:

(3) G(t)=1+1〈N〉(1+Ftrip1−Ftripe−τTtrip)∑i=1nFi(1+(tτdiff,i))1+(ωxyωz)2(tτdiff,i)

where 〈N〉 represents the average number of fluorescent particles in the confocal volume. The exponential term describes the triplet state behavior of the molecule, in which *F_trip_* is the fraction of molecules in the triplet state and *T_trip_* is the average time a molecule resides in the triplet state. The last part of the equation describes the diffusion behavior of the molecules, where *F_i_* is the fraction of species *i*, τ_diff,i_ is the diffusion time of species *i*, ω_xy_ and ω_z_ are the equatorial and axial radii of the detection volume, respectively. Equation (3) was used to obtain 〈N〉 for the different samples.

### 4.9. Steady-State Fluorescence Spectroscopy

Fluorescence excitation and emission spectra were measured using a Cary Eclipse spectrofluorimeter (Varian). Excitation and emission slits were set to yield bandwidths of 5 nm. All measurements were performed at 20 °C. Samples with concentrations of 1 μM FP were measured free in buffered solution as well as encapsulated with P2MVP_41_-*b*-PEO_205_ or P2MVP_128_-*b*-PEO_477_ at their respective PMCs.

The relative quantum yields are calculated using the following equation [69]:

(4) $${\mathrm{QY}}_{C3M}=\frac{{\mathrm{QY}}_{P}\cdot {\mathrm{FA}}_{C3M}\cdot A_{P}}{{\mathrm{FA}}_{P}\cdot A_{C3M}}$$

where QY represents the quantum yield, FA the integrated area under the corrected emission spectrum, and A the absorbance at the excitation wavelength. The subscripts *C3M* and *P* refer to the proteins in the C3Ms and the proteins free in solution, respectively.

### 4.10. Circular Dichroism (CD)

CD experiments were performed on a JASCO J-715 spectropolarimeter with a Jasco PTC 348 WI temperature controller set at 20 °C. The far-UV CD spectra (195–260 nm) were obtained from samples in a 0.3 mL quartz cuvette with an optical path length of 1 mm. Thirty spectra, each recorded with a resolution of 1 nm, a scan speed of 50 nm/min and a response time of 1 s, were accumulated and averaged. Samples with concentrations of 2.5 μM FP were measured free in buffered solution as well as encapsulated with P2MVP_41_-*b*-PEO_205_ or P2MVP_128_-*b*-PEO_477_ at their respective PMC. The polymers did not show any CD signal over the measured range, therefore, buffer blank spectra, obtained at identical conditions, were subtracted.

## 5. Conclusions

We have studied the encapsulation efficiency of SBFP2, mTurquoise2, mEGFP, SYFP2, mKO2, TagRFP, and mCherry and determined their spectral and structural properties as protein free in solution and upon encapsulation with P2MVP_41_-*b*-PEO_205_ or P2MVP_128_-*b*-PEO_477_. This revealed that *av*FPs are almost 100% encapsulated, while *an*FPs show encapsulation efficiencies ranging between 50% and 75%. Upon encapsulation, all FPs show differences in spectral properties compared to their respective protein free in solution: the chromophores of SBFP2, mKO2, and mCherry are affected in their molar extinction coefficient and the chromophores of mTurquoise2, mEGFP, SYFP2, TagRFP, and mKO2 are affected in their fluorescence quantum yield. Only for mCherry, the changes in spectral properties upon encapsulation are similar to changes observed as a result of pH variation and are, therefore, related to a shift in the p*K*_a_. Even though all FPs have an 11-stranded β-barrel fold, the CD spectra differ between *av*FPs and *an*FPs. This is most likely due to a different shape of the cylinders between the two groups of FPs, where the β-barrel structures of *av*FPs are almost round cylinders and that of *an*FPs elliptic ones. This variation in structure, together with the difference in charge distribution on FP surfaces, potentially causes the differences in encapsulation efficiency.

## Figures and Tables

**Table 1:** Results of PROPKA 3.1 analysis and preferred micellar composition (PMC) determination. The charge of the proteins was determined at pH 9, except for TagRFP (pH 10), with PROPKA 3.1 [32,33]. PMC (*F*^+^) and hydrodynamic radii (with standard deviations) were determined with dynamic light scattering for all used fluorescent protein variants encapsulated using the two diblock copolymers.

Fluorescent Protein Variant	Charge	P2MVP_41_-*b*-PEO_205_		P2MVP_128_-*b*-PEO_477_	
		PMC (*F*^+^)	Radius (nm)	PMC (*F*^+^)	Radius (nm)
SBFP2	−8.96	0.75	38.3 ± 0.5	0.70	33.6 ± 0.2
mTurquoise2	−11.30	0.70	37.1 ± 1.0	0.70	33.9 ± 0.5
mEGFP	−9.87	0.70	30.4 ± 0.5	0.65	32.2 ± 0.2
SYFP2	−9.75	0.70	30.4 ± 0.4	0.60	35.2 ± 0.7
mKO2	−13.09	0.65	25.7 ± 0.5	0.60	28.3 ± 0.5
TagRFP ^a^	−10.35	0.70	36.7 ± 5.0	0.65	33.3 ± 1.9
mCherry	−8.93	0.75	30.0 ± 0.8	0.75	35.1 ± 1.0

^a^ Values determined at pH 10.

**Table 2:** Quantum yields of the encapsulated proteins in C3Ms with P2MVP_41_-*b*-PEO_205_ (C3M_P41) and P2MVP_128_-*b*-PEO_477_ (C3M_P128) compared to those of the proteins free in solution according to Equation (4).

Fluorescent Protein Variant	Free Protein	C3M_P41	C3M_P128
SBFP2	0.47	0.47	0.46
mTurquoise2	0.93	0.84	0.88
mEGFP	0.60	0.44	0.50
SYFP2	0.68	0.60	0.62
mKO2	0.62	0.41	0.45
TagRFP	0.48	0.33	0.25
mCherry	0.22	0.25	0.24

**Table 5:** Settings for the FCS measurements per studied protein.

Protein	Laser Line (nm)	Spectral Filter (nm)
mTurquoise2	440	475–500
mEGFP	488	495–525
SYFP2	514	520–550
mKO2	550	650–600
TagRFP and mCherry	561	575–610

## Supplementary Materials

References [70,71,72,73,74] appear in the Supplementary Materials. Supplementary materials can be found at www.mdpi.com/1422-0067/18/7/1557/s1.

## Abbreviations

*an*FP	Anthozoa fluorescent protein
*av*FP	*Aequorea victoria* fluorescent protein
C3M	Complex coacervate core micelles
CD	Circular dichroism
DLS	Dynamic light scattering
EGFP	Enhanced green fluorescent protein
FCS	Fluorescence correlation spectroscopy
FP	Fluorescent protein
FRET	Förster resonance energy transfer
GFP	Green fluorescent protein
mEGFP	Monomeric enhanced green fluorescent protein
mKO2	Monomeric Kusabira Orange 2
P128	P2MVP_128_-*b*-PEO_477_
P2MVP-*b*-PEO	poly(2-methyl-vinyl-pyridinium)-*b*-poly(ethylene-oxide)
P41	P2MVP_41_-*b*-PEO_205_
PDB	Protein Data Bank
PMC	Preferred micellar composition
SBFP2	Strongly enhanced blue fluorescent protein 2
SYFP2	Strongly enhanced yellow fluorescent protein 2
TagRFP	Tag red fluorescent protein
