# Supplementary material for: Colorful Packages: Encapsulation of Fluorescent Proteins in Complex Coacervate Core Micelles
Source: Int J Mol Sci. 2017 Jul 19;18(7):1557. doi: 10.3390/ijms18071557 (PMC5536045; doi:10.3390/ijms18071557)
Supplement: Supplementary file 1 [file ijms-18-01557-s001.pdf]

Int. J. Mol. Sci. **2017**, *18*, 1557; doi:10.3390/ijms18071557

Supplementary Materials to

Colorful Packages; Encapsulation of Fluorescent Proteins in Complex Coacervate Core Micelles

Antsje Nolles, Adrie H. Westphal, J. Mieke Kleijn, Willem J. H. van Berkel and Jan Willem Borst

Index

1. Sequence Identities and Multiple Structural Alignment of the Studied Fluorescent Proteins 2

2. Fluorescent Protein Characteristics 3

3. Dynamic Light Scattering Results 4

4. Fluorescence Correlation Spectroscopy Results 5

5. Absorption Spectral Analysis 6

6. Steady-State Fluorescence at Different pH Values 7

7. High Tension Graphs Related to the Far-UV CD Spectra 8

8. Sequence Alignment of All Fluorescent Proteins with Their Protein Data Bank Entries 9

9. References 13

1. Sequence Identities and Multiple Structural Alignment of the Studied Fluorescent Proteins

Table S1. Sequence identity percentages (%) between pairs of fluorescent proteins (FP variant) that have been studied.

FP variant	mEGFP	SBFP2	mTurquoise2	SYFP2	mKO2	TagRFP	mCherry
**mEGFP**	100	97.1	96.7	97.1	27.5	25.3	29.5
**SBFP2**	97.1	100	98.3	97.9	27.5	25.3	29.5
**mTurquoise2**	96.7	98.3	100	97.5	27.5	24.9	29.5
**SYFP2**	97.1	97.9	97.5	100	28.4	25.8	30.4
**mKO2**	27.5	27.5	27.5	28.4	100	47.1	48.5
**TagRFP**	25.3	25.3	24.9	25.8	47.1	100	56.9
**mCherry**	29.5	29.5	29.5	30.4	48.5	56.9	100

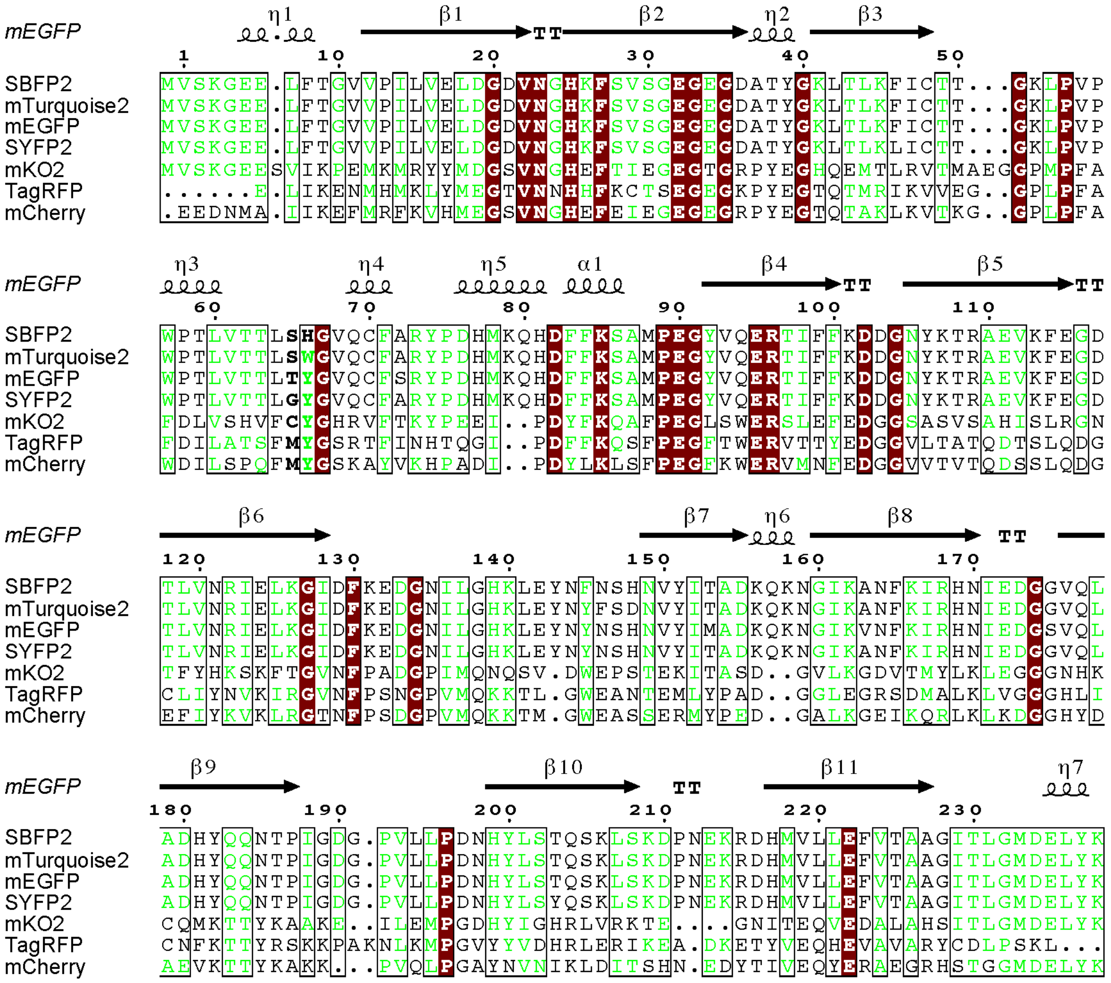

Figure S1. Multiple structural alignment of seven fluorescent proteins that were studied. Protein structures were aligned using msTALI software [1]. The alignment was then manually adjusted and drawn using ESPript 3.0 software [2]. Strictly conserved amino acid residues are shown with brown background and similar amino acid residues are boxed and shown in green. The amino acid residues forming the chromophore are indicated in bold letters. Secondary structure elements derived from mEGFP (PDB entry 4EUL [3]) are depicted as arrows (representing β-strands), coils (representing α- and 3_{10}-helices), and TT letters (representing turns). The numbering is based on that of mEGFP.

2. Fluorescent Protein Characteristics

Table S2 shows the characteristics of the studied fluorescent proteins (FPs). Next to the standard characteristics, some specific features for this article are given, *i.e.*, charge, monomeric quality and dissociation constant. The net charge of the FPs at pH 9.0 or pH 10.0 is given, which were calculated using the software package PROPKA 3.1 [4, 5]. The monomeric qualities of more than 40 FPs were determined by Cranfill, *et al*. [6] For this, they fused FPs onto an endoplasmic reticulum (ER) membrane protein (CytERM). If the FP formed homo-oligomers due to high effective concentrations, the ER configured from a tubular network into an organized smooth ER whorl structure. The percentage of observed cells exhibiting an organized smooth ER whorl structure was related to the monomeric quality of the FPs. The dissociation constants were determined by sedimentation equilibrium analytical ultracentrifugation experiments. The A206K mutation introduced into yellow fluorescent protein (YFP) increased the dissociation constant from 0.11 to 74 mM [7]. This mutation is present in SBFP2, mTurquoise2, mEGFP, and SYFP2, providing these proteins with dissociation constants of about 74 mM. Next to that, mTurquoise2 bears the N146F mutation resulting in an increased dissociation constant [8]. The K_D of mKO2 is not determined so far. The dissociation constants of TagRFP and mCherry were investigated by Han, *et al*. [9]. For TagRFP a K_D of 0.038 mM was found and the K_D of mCherry was beyond the limit of their instrument.

Table S2. Properties of the studied fluorescent proteins (FP variant).

FP variant	λ_{ex} (nm)	λ_{em} (nm)	EC (M^{-1} cm^{-1})	QY	pK_a	pI	Charge[c]	Monomeric quality (%)[e]	K_D (mM)	Reference
SBFP2	380	446	34000	0.47	5.5	5.59	-8.96	nd	74.0[f,g]	Kremers, et al. [10]
mTurquoise2	434	474	30000	0.93	3.1	5.29	-11.30	93.8	> 74.0[f,g,h]	Goedhart, et al. [11]
mEGFP	488	507	56000	0.60	6.0	5.49	-9.87	98.1	74.0[f,g]	Yang, et al. [12]
SYFP2	515	527	101000	0.68	6.0	5.62	-9.75	nd	74.0[f,g]	Kremers, et al. [13]
mKO2	551	565	63800	0.62	5.5	5.48	-13.09	68.4	nd	Sakaue-Sawano, et al. [14]
TagRFP	555	584	100000	0.48	3.8	7.43	-10.35[d]	57.7	0.038[i]	Merzlyak, et al. [15]
mCherry	587	610	72000	0.22	4.5[a], 10.3[b]	5.70	-8.93	95.0	> 0.050[i]	Shaner, et al. [16]

[a]from Shaner, et al. [16], [b]from Shu, et al. [17], [c]Charge based on PROPKA 3.1 results, determined at their respective pH value used for the experiments, [d]Value determined at pH 10, [e]from Cranfill, et al. [6], [f]from Zacharias, et al. [7], [g]Value based on the presence of the A206K mutation, [h]from von Stetten, et al. [8], [i]from Han, et al. [9], nd, not determined.

3. Dynamic Light Scattering Results

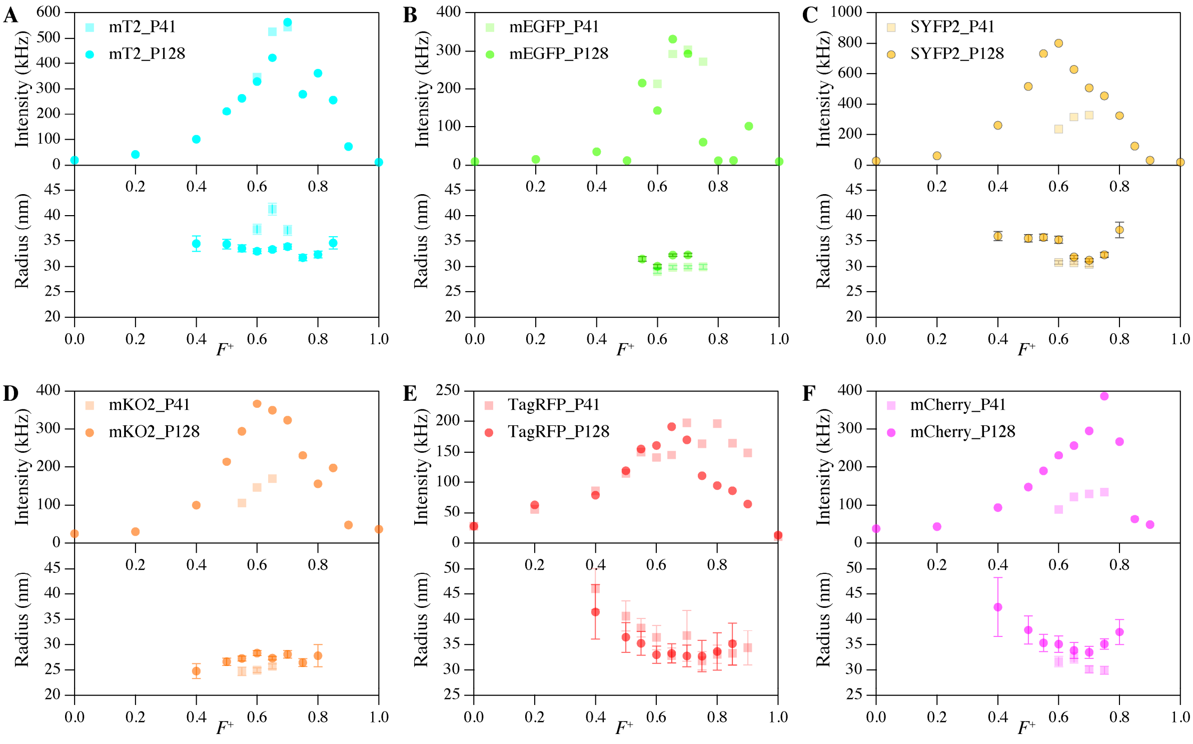

Figure S2. Dynamic light scattering composition results of (A) mTurquoise2 (mT2), (B) mEGFP, (C) SYFP2, (D) mKO2, (E) TagRFP, and (F) mCherry with $P2MVP_{41}$-*b*-PEO_{205} (P41, light colored blocks) and $P2MVP_{128}$-*b*-PEO_{477} (P128, dark colored circles) wherein the concentration of protein was kept constant. Top graphs show scattered intensity as a function of the F^+ composition, and bottom graphs show hydrodynamic radius as a function of the F^+ composition. Error bars show the distribution of radii in one experiment.

4. Fluorescence Correlation Spectroscopy Results

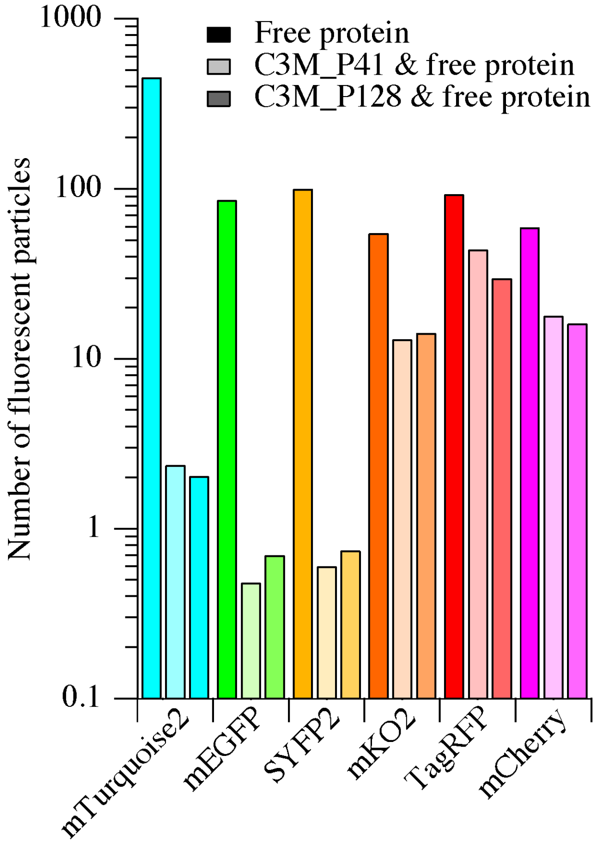

Figure S3. Fluorescence correlation spectroscopy results the number of fluorescent particles measured of all used FPs (except SBFP2) free in solution (darkest colored, left bars), and measured at the PMC with $P2MVP_{41}$-*b*-PEO_{205} (C3M_P41, light colored, middle bars) and $P2MVP_{128}$-*b*-PEO_{477} (C3M_P128, dark colored, right bars).

5. Absorption Spectral Analysis

Absorption spectra were recorded on a Hewlett Packard 8453 diode array spectrophotometer in 10 mM borate buffer at pH 9.0 for SBFP2, mTurquoise2, mEGFP, SYFP2, mKO2, and mCherry and at pH 10.0 for TagRFP at 20°C. Spectrophotometer settings were controlled using the UV-Visible ChemStation software package (Hewlett Packard, Palo Alto, CA, USA). Samples with concentrations of 1 μM FP were measured free in buffered solution as well as encapsulated with $P2MVP_{41}$-*b*-PEO_{205} and $P2MVP_{128}$-*b*-PEO_{477} at their respective PMCs.

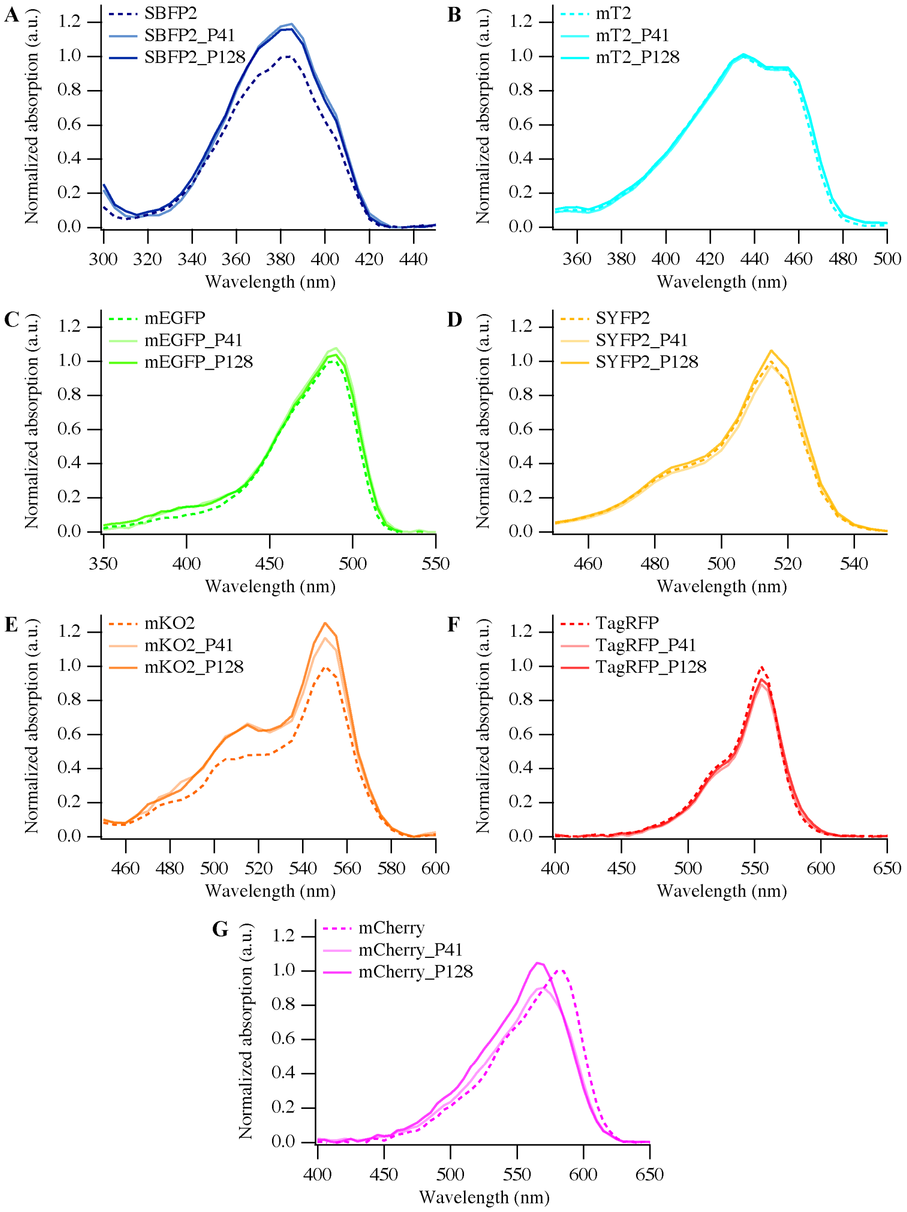

Figure S4. Normalized absorption spectra of (A) SBFP2, (B) mTurqouise2 (mT2), (C) mEGFP, (D) SYFP2, (E) mKO2, (F) TagRFP, and (G) mCherry for proteins free in solution (dashed lines) and encapsulated proteins in C3Ms at their respective PMCs with $P2MVP_{41}$-*b*-PEO_{205} (P41, solid light colored line) and $P2MVP_{128}$-*b*-PEO_{477} (P128, solid dark colored line). The spectra are normalized to those of the free proteins.

6. Steady-State Fluorescence at Different pH Values

Fluorescence excitation and emission spectra were measured using a Cary Eclipse spectrofluorimeter (Varian). Excitation and emission slits were set to yield bandwidths of 5 nm. All measurements were performed at 20°C. A master buffer was used consisting of 20 mM sodium phosphate, 20 mM citric acid, 10 mM glycine, and 150 mM NaCl adjusted to the desired pH by addition of NaOH. Samples with concentrations of 1 µM FP at pH 5.2, 7.1, 9.0, and 10.0 were measured.

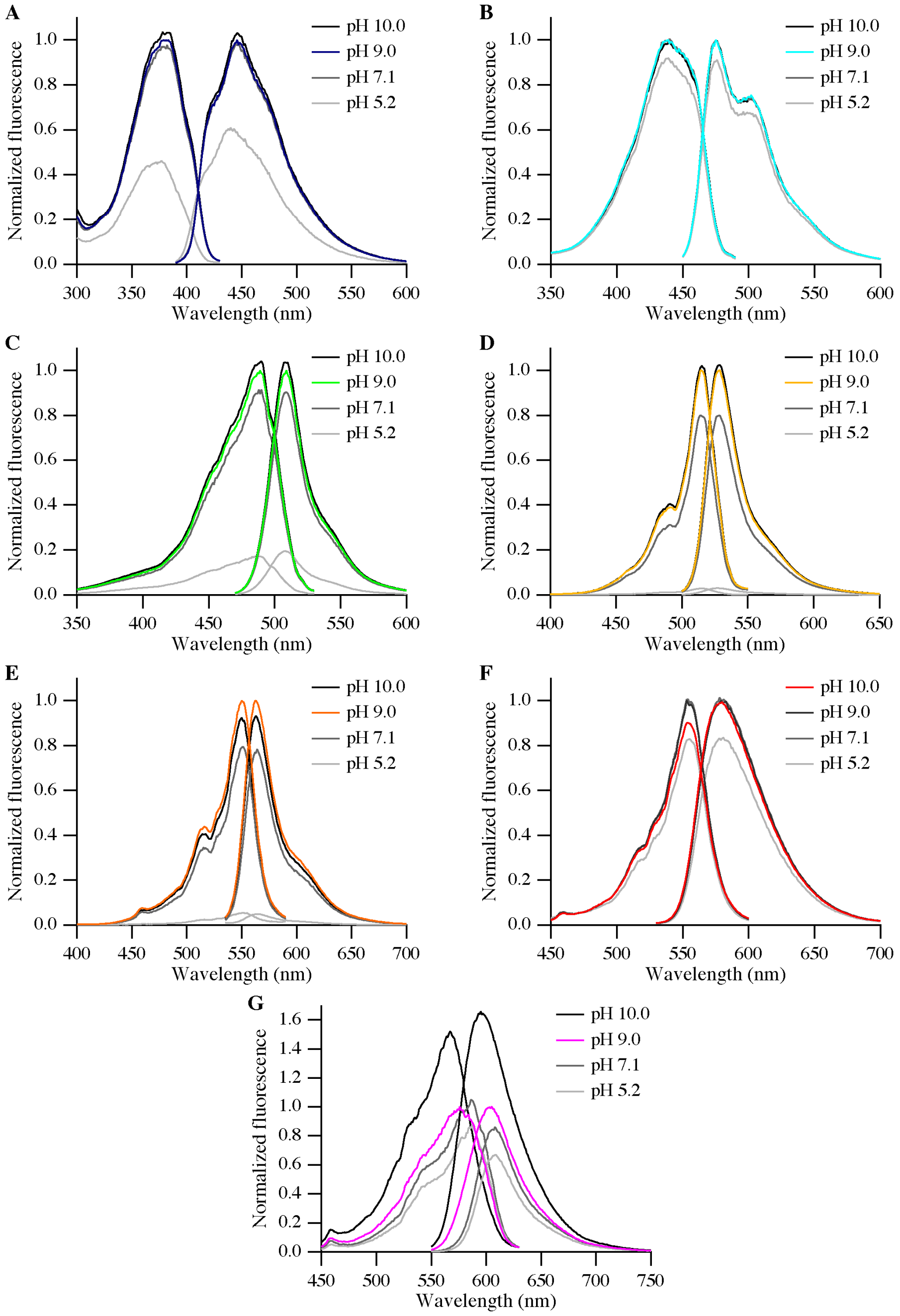

Figure S5. Normalized fluorescence excitation and emission spectra of (A) SBFP2, (B) mTurqouise2, (C) mEGFP, (D) SYFP2, (E) mKO2, (F) TagRFP, and (G) mCherry in solutions with different pH values. The spectra are colored according to the FP at the respective pH: pH 9.0 for SBFP2, mTurqouise2, mEGFP, SYFP2, mKO2, and mCherry and pH 10.0 for TagRFP. Spectra are normalized to the FPs at pH 9.0.

7. High Tension Graphs Related to the Far-UV CD Spectra

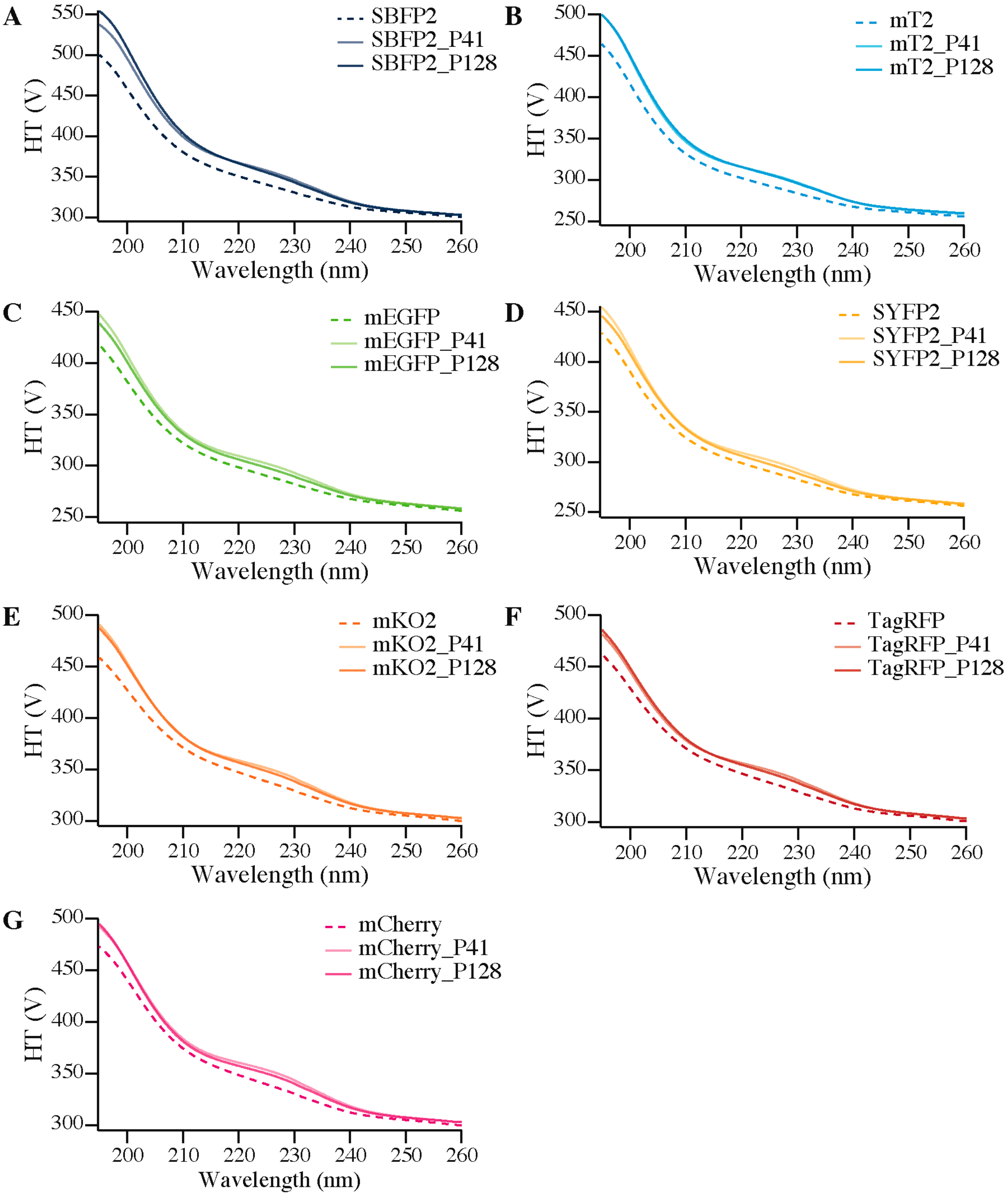

Figure S6. High tension (HT) signals of free fluorescent proteins (dashed lines) and encapsulated with $P2MVP_{41}$-*b*-PEO_{205} (P41, solid light colored line) and $P2MVP_{128}$-*b*-PEO_{477} (P128, solid dark colored line) belonging to the far-UV CD spectra in Figure 6: (A) SBFP2, (B) mTurquoise2 (mT2), (C) mEGFP, (D) SYFP2, (E) mKO2, (F) TagRFP, and (G) mCherry.

8. Sequence Alignment of All Fluorescent Proteins with Their Protein Data Bank Entries

```
                10        20        30        40        50        60
SBFP2    VSKGEELFTGVVPILVELDGDVNGHKFSVSGEGEGDATYGKLTLKFICTTGKLPVPWPTL
         .:::::::::::::::::::::::::::::::::::::::::::::::::::::::::::
1BFP     MSKGEELFTGVVPILVELDGDVNGHKFSVSGEGEGDATYGKLTLKFICTTGKLPVPWPTL
                 10        20        30        40        50        60

                70        80        90       100       110       120
SBFP2    VTTLSHGVQCFARYPDHMKQHDFFKSAMPEGYVQERTIFFKDDGNYKTRAEVKFEGDTLV
         :::.:::::::.:::::::.::::::::::::::::::::::::::::::::::::::::
1BFP     VTTFSHGVQCFSRYPDHMKRHDFFKSAMPEGYVQERTIFFKDDGNYKTRAEVKFEGDTLV
                 70        80        90       100       110       120

               130       140       150       160       170       180
SBFP2    NRIELKGIDFKEDGNILGHKLEYNFNSHNVYITADKQKNGIKANFKIRHNIEDGGVQLAD
         :::::::::::::::::::::::::::::::: :::::::::.:::::::::::.:::::
1BFP     NRIELKGIDFKEDGNILGHKLEYNFNSHNVYIMADKQKNGIKVNFKIRHNIEDGSVQLAD
                130       140       150       160       170       180

               190       200       210       220       230
SBFP2    HYQQNTPIGDGPVLLPDNHYLSTQSKLSKDPNEKRDHMVLLEFVTAAGITLGMDELYK
         ::::::::::::::::::::::::: :::::::::::::::::::::::: :::::::
1BFP     HYQQNTPIGDGPVLLPDNHYLSTQSALSKDPNEKRDHMVLLEFVTAAGITHGMDELYK
                190       200       210       220       230
```

Figure S7. Pairwise sequence alignment of SBFP2 with the template 1BFP generated by lalign [18]. The identical (double points) and similar (single point) residues are highlighted.

```
                 10        20        30        40        50        60
mT2      MVSKGEELFTGVVPILVELDGDVNGHKFSVSGEGEGDATYGKLTLKFICTTGKLPVPWPT
         ::::::::::::::::::::::::::::::::::::::::::::::::::::::::::::
3ZTF     MVSKGEELFTGVVPILVELDGDVNGHKFSVSGEGEGDATYGKLTLKFICTTGKLPVPWPT
                 10        20        30        40        50        60

                 70        80        90       100       110       120
mT2      LVTTLSWGVQCFARYPDHMKQHDFFKSAMPEGYVQERTIFFKDDGNYKTRAEVKFEGDTL
         ::::::::::::::::::::::::::::::::::::::::::::::::::::::::::::
3ZTF     LVTTLSWGVQCFARYPDHMKQHDFFKSAMPEGYVQERTIFFKDDGNYKTRAEVKFEGDTL
                 70        80        90       100       110       120

                130       140       150       160       170       180
mT2      VNRIELKGIDFKEDGNILGHKLEYNYFSDNVYITADKQKNGIKANFKIRHNIEDGGVQLA
         ::::::::::::::::::::::::::::::::::::::::::::::::::::::::::::
3ZTF     VNRIELKGIDFKEDGNILGHKLEYNYFSDNVYITADKQKNGIKANFKIRHNIEDGGVQLA
                130       140       150       160       170       180

                190       200       210       220       230
mT2      DHYQQNTPIGDGPVLLPDNHYLSTQSKLSKDPNEKRDHMVLLEFVTAAGITLGMDELYK
         :::::::::::::::::::::::::: ::::::::::::::::::::::::::::::::
3ZTF     DHYQQNTPIGDGPVLLPDNHYLSTQSALSKDPNEKRDHMVLLEFVTAAGITLGMDELYK
                190       200       210       220       230
```

Figure S8. Pairwise sequence alignment of mTurquoise2 (mT2) with the template 3ZTF generated by lalign [18]. The identical (double points) and similar (single point) residues are highlighted.

```
               10        20        30        40        50        60
mEGFP  MVSKGEELFTGVVPILVELDGDVNGHKFSVSGEGEGDATYGKLTLKFICTTGKLPVPWPT
       ::::::::::::::::::::::::::::::::::::::::::::::::::::::::::::
4EUL   MVSKGEELFTGVVPILVELDGDVNGHKFSVSGEGEGDATYGKLTLKFICTTGKLPVPWPT
               10        20        30        40        50        60

               70        80        90       100       110       120
mEGFP  LVTTLTYGVQCFSRYPDHMKQHDFFKSAMPEGYVQERTIFFKDDGNYKTRAEVKFEGDTL
       ::::::::::::::::::::::::::::::::::::::::::::::::::::::::::::
4EUL   LVTTLTYGVQCFSRYPDHMKQHDFFKSAMPEGYVQERTIFFKDDGNYKTRAEVKFEGDTL
               70        80        90       100       110       120

              130       140       150       160       170       180
mEGFP  VNRIELKGIDFKEDGNILGHKLEYNYNSHNVYIMADKQKNGIKVNFKIRHNIEDGSVQLA
       ::::::::::::::::::::::::::::::::::::::::::::::::::::::::::::
4EUL   VNRIELKGIDFKEDGNILGHKLEYNYNSHNVYIMADKQKNGIKVNFKIRHNIEDGSVQLA
              130       140       150       160       170       180

              190       200       210       220       230
mEGFP  DHYQQNTPIGDGPVLLPDNHYLSTQSKLSKDPNEKRDHMVLLEFVTAAGITLGMDELYK
       :::::::::::::::::::::::::: :::::::::::::::::::::::::::::::
4EUL   DHYQQNTPIGDGPVLLPDNHYLSTQSALSKDPNEKRDHMVLLEFVTAAGITLGMDELYK
              190       200       210       220       230
```

Figure S9. Pairwise sequence alignment of mEGFP with the template 4EUL generated by lalign [18]. The identical (double points) and similar (single point) residues are highlighted.

```
               10        20        30        40        50        60
SYFP2  MVSKGEELFTGVVPILVELDGDVNGHKFSVSGEGEGDATYGKLTLKLICTTGKLPVPWPT
       ::::::::::::::::::::::::::::::::::::::::::::::::::::::::::::
1MYW   MVSKGEELFTGVVPILVELDGDVNGHKFSVSGEGEGDATYGKLTLKLICTTGKLPVPWPT
               10        20        30        40        50        60

               70        80        90       100       110       120
SYFP2  LVTTLGYGVQCFARYPDHMKQHDFFKSAMPEGYVQERTIFFKDDGNYKTRAEVKFEGDTL
       ::::::::.:::::::::::::::::::::::::::::::::::::::::::::::::::
1MYW   LVTTLGYGLQCFARYPDHMKQHDFFKSAMPEGYVQERTIFFKDDGNYKTRAEVKFEGDTL
               70        80        90       100       110       120

              130       140       150       160       170       180
SYFP2  VNRIELKGIDFKEDGNILGHKLEYNYNSHNVYITADKQKNGIKANFKIRHNIEDGGVQLA
       ::::::::::::::::::::::::::::::::::::::::::::::::::::::::::::
1MYW   VNRIELKGIDFKEDGNILGHKLEYNYNSHNVYITADKQKNGIKANFKIRHNIEDGGVQLA
              130       140       150       160       170       180

              190       200       210       220       230
SYFP2  DHYQQNTPIGDGPVLLPDNHYLSYQSKLSKDPNEKRDHMVLLEFVTAAGITLGMDELYK
       :::::::::::::::::::::::::: ::::::::::::::::::::::: :::::::
1MYW   DHYQQNTPIGDGPVLLPDNHYLSYQSALSKDPNEKRDHMVLLEFVTAAGITHGMDELYK
              190       200       210       220       230
```

Figure S10. Pairwise sequence alignment of SYFP2 with the template 1MYW generated by lalign [18]. The identical (double points) and similar (single point) residues are highlighted.

```
         10        20        30        40        50        60
mKO2    SVIKPEMKMRYYMDGSVNGHEFTIEGEGTGRPYEGHQEMTLRVTMAEGGPMPFAFDLVSH
        ::::::::::::::::::::::::::::::::::::::::::::::.:::::::::::::
2ZMU    SVIKPEMKMRYYMDGSVNGHEFTIEGEGTGRPYEGHQEMTLRVTMAKGGPMPFAFDLVSH
              10        20        30        40        50        60

         70        80        90       100       110       120
mKO2    VFCYGHRVFTKYPEEIPDYFKQAFPEGLSWERSLEFEDGGSASVSAHISLRGNTFYHKSK
        ::::::: ::::::::::::::::::::::::::::::::::::::::::::::::::::
2ZMU    VFCYGHRPFTKYPEEIPDYFKQAFPEGLSWERSLEFEDGGSASVSAHISLRGNTFYHKSK
              70        80        90       100       110       120

        130       140       150       160       170       180
mKO2    FTGVNFPADGPIMQNQSVDWEPSTEKITASDGVLKGDVTMYLKLEGGGNHKCQMKTTYKA
        :::::::::::::::::::::::::::::::::::::::::::::::::::::.::::::
2ZMU    FTGVNFPADGPIMQNQSVDWEPSTEKITASDGVLKGDVTMYLKLEGGGNHKCQFKTTYKA
             130       140       150       160       170       180

        190       200       210       220
mKO2    AKEILEMPGDHYIGHRLVRKTEGNITEQVEDALAHS
        ::.::.:::.:::.::::::::::::: ::::.:::
2ZMU    AKKILKMPGSHYISHRLVRKTEGNITELVEDAVAHS
             190       200       210
```

Figure S11. Pairwise sequence alignment of mKO2 with the template 2ZMU generated by lalign [18]. The identical (double points) and similar (single point) residues are highlighted.

```
                10        20        30        40        50        60
TagRFP  MVSKGEELIKENMHMKLYMEGTVNNHHFKCTSEGEGKPYEGTQTMRIKVVEGGPLPFAFD
        ::::::::::::::::::::::::::::::::::::::::::::::::::::::::::::
3M22    MVSKGEELIKENMHMKLYMEGTVNNHHFKCTSEGEGKPYEGTQTMRIKVVEGGPLPFAFD
                10        20        30        40        50        60

                70        80        90       100       110       120
TagRFP  ILATSFMYGSRTFINHTQGIPDFFKQSFPEGFTWERVTTYEDGGVLTATQDTSLQDGCLI
        ::::::::::::::::::::::::::::::::::::::::::::::::::::::::::::
3M22    ILATSFMYGSRTFINHTQGIPDFFKQSFPEGFTWERVTTYEDGGVLTATQDTSLQDGCLI
                70        80        90       100       110       120

               130       140       150       160       170       180
TagRFP  YNVKIRGVNFPSNGPVMQKKTLGWEANTEMLYPADGGLEGRSDMALKLVGGGHLICNFKT
        ::::::::::::::::::::::::::::::::::::::::::::::::::::::::::::
3M22    YNVKIRGVNFPSNGPVMQKKTLGWEANTEMLYPADGGLEGRSDMALKLVGGGHLICNFKT
               130       140       150       160       170       180

               190       200       210       220       230
TagRFP  TYRSKKPAKNLKMPGVYYVDHRLERIKEADKETYVEQHEVAVARYCDLPSKLLYK
        :::::::::::::::::::::::::::::::::::::::::::::::::::: .:
3M22    TYRSKKPAKNLKMPGVYYVDHRLERIKEADKETYVEQHEVAVARYCDLPSKLGHK
               190       200       210       220       230
```

Figure S12. Pairwise sequence alignment of TagRFP with the template 3M22 generated by lalign [18]. The identical (double points) and similar (single point) residues are highlighted.

```
                 10        20        30        40        50        60
mCherry MVSKGEEDNMAIIKEFMRFKVHMEGSVNGHEFEIEGEGEGRPYEGTQTAKLKVTKGGPLP
        ::::::::::::::::::::::::::::::::::::::::::::::::::::::::::::
2H5Q    MVSKGEEDNMAIIKEFMRFKVHMEGSVNGHEFEIEGEGEGRPYEGTQTAKLKVTKGGPLP
                 10        20        30        40        50        60

                 70        80        90       100       110       120
mCherry FAWDILSPQFMYGSKAYVKHPADIPDYLKLSFPEGFKWERVMNFEDGGVVTVTQDSSLQD
        ::::::::::::::::::::::::::::::::::::::::::::::::::::::::::::
2H5Q    FAWDILSPQFMYGSKAYVKHPADIPDYLKLSFPEGFKWERVMNFEDGGVVTVTQDSSLQD
                 70        80        90       100       110       120

                130       140       150       160       170       180
mCherry GEFIYKVKLRGTNFPSDGPVMQKKTMGWEASSERMYPEDGALKGEIKQRLKLKDGGHYDA
        ::::::::::::::::::::::::::::::::::::::::::::::::::::::::::::
2H5Q    GEFIYKVKLRGTNFPSDGPVMQKKTMGWEASSERMYPEDGALKGEIKQRLKLKDGGHYDA
                130       140       150       160       170       180

                190       200       210       220       230
mCherry EVKTTYKAKKPVQLPGAYNVNIKLDITSHNEDYTIVEQYERAEGRHSTGGMDELYK
        ::::::::::::::::::::::::::::::::::::::::::::::::::::::::
2H5Q    EVKTTYKAKKPVQLPGAYNVNIKLDITSHNEDYTIVEQYERAEGRHSTGGMDELYK
                190       200       210       220       230
```

Figure S13. Pairwise sequence alignment of mCherry with the template 2H5Q generated by lalign [18]. The identical (double points) and similar (single point) residues are highlighted.

9. References

1. Shealy, P.; Valafar, H., Multiple structure alignment with msTALI. *BMC Bioinf.* **2012,** 13, 105.
2. Robert, X.; Gouet, P., Deciphering key features in protein structures with the new ENDscript server. *Nucleic Acids Res.* **2014,** 42, (Web Server issue), W320-324.
3. Arpino, J. A.; Rizkallah, P. J.; Jones, D. D., Crystal structure of enhanced green fluorescent protein to 1.35 A resolution reveals alternative conformations for Glu222. *PLoS ONE* **2012,** 7, e47132.
4. Rostkowski, M.; Olsson, M. H. M.; Søndergaard, C. R.; Jensen, J. H., Graphical analysis of pH-dependent properties of proteins predicted using PROPKA. *BMC Struct. Biol.* **2011,** 11, 6.
5. Olsson, M. H.; Søndergaard, C. R.; Rostkowski, M.; Jensen, J. H., PROPKA3: Consistent treatment of internal and surface residues in empirical pKa predictions. *J. Chem. Theory Comput.* **2011,** 7, 525-537.
6. Cranfill, P. J.; Sell, B. R.; Baird, M. A.; Allen, J. R.; Lavagnino, Z.; de Gruiter, H. M.; Kremers, G. J.; Davidson, M. W.; Ustione, A.; Piston, D. W., Quantitative assessment of fluorescent proteins. *Nat. Methods* **2016,** 13, 557-562.
7. Zacharias, D. A.; Violin, J. D.; Newton, A. C.; Tsien, R. Y., Partitioning of lipid-modified monomeric GFPs into membrane microdomains of live cells. *Science* **2002,** 296, 913-916.
8. von Stetten, D.; Noirclerc-Savoye, M.; Goedhart, J.; Gadella, T. W., Jr.; Royant, A., Structure of a fluorescent protein from *Aequorea victoria* bearing the obligate-monomer mutation A206K. *Acta Crystallogr., Sect. F: Struct. Biol. Cryst. Commun.* **2012,** 68, 878-882.
9. Han, L.; Zhao, Y.; Zhang, X.; Peng, J.; Xu, P.; Huan, S.; Zhang, M., RFP tags for labeling secretory pathway proteins. *Biochem. Biophys. Res. Commun.* **2014,** 447, 508-512.
10. Kremers, G. J.; Goedhart, J.; van den Heuvel, D. J.; Gerritsen, H. C.; Gadella, T. W. J., Improved green and blue fluorescent proteins for expression in bacteria and mammalian cells. *Biochemistry* **2007,** 46, 3775-3783.
11. Goedhart, J.; von Stetten, D.; Noirclerc-Savoye, M.; Lelimousin, M.; Joosen, L.; Hink, M. A.; van Weeren, L.; Gadella, T. W. J.; Royant, A., Structure-guided evolution of cyan fluorescent proteins towards a quantum yield of 93%. *Nat. Commun.* **2012,** 3, 751.
12. Yang, T. T.; Cheng, L.; Kain, S. R., Optimized codon usage and chromophore mutations provide enhanced sensitivity with the green fluorescent protein. *Nucleic Acids Res.* **1996,** 24, 4592-4593.
13. Kremers, G. J.; Goedhart, J.; van Munster, E. B.; Gadella, T. W. J., Cyan and yellow super fluorescent proteins with improved brightness, protein folding, and FRET Forster radius. *Biochemistry* **2006,** 45, 6570-6580.
14. Sakaue-Sawano, A.; Kurokawa, H.; Morimura, T.; Hanyu, A.; Hama, H.; Osawa, H.; Kashiwagi, S.; Fukami, K.; Miyata, T.; Miyoshi, H.; Imamura, T.; Ogawa, M.; Masai, H.; Miyawaki, A., Visualizing spatiotemporal dynamics of multicellular cell-cycle progression. *Cell* **2008,** 132, 487-498.
15. Merzlyak, E. M.; Goedhart, J.; Shcherbo, D.; Bulina, M. E.; Shcheglov, A. S.; Fradkov, A. F.; Gaintzeva, A.; Lukyanov, K. A.; Lukyanov, S.; Gadella, T. W. J.; Chudakov, D. M., Bright monomeric red fluorescent protein with an extended fluorescence lifetime. *Nat. Methods* **2007,** 4, 555-557.
16. Shaner, N. C.; Campbell, R. E.; Steinbach, P. A.; Giepmans, B. N.; Palmer, A. E.; Tsien, R. Y., Improved monomeric red, orange and yellow fluorescent proteins derived from *Discosoma sp.* red fluorescent protein. *Nat. Biotechnol.* **2004,** 22, 1567-1572.
17. Shu, X.; Shaner, N. C.; Yarbrough, C. A.; Tsien, R. Y.; Remington, S. J., Novel chromophores and buried charges control color in mFruits. *Biochemistry* **2006,** 45, 9639-9647.
18. Huang, X. Q.; Miller, W., A time-efficient, linear-space local similarity algorithm. *Adv. Appl. Math.* **1991,** 12, 337-357.
